# *KIR AA* individuals possess strong inhibitory *KIR* alleles alongside HLA ligands that are protective against leukemia in the Chinese population

**DOI:** 10.3389/fgene.2025.1745482

**Published:** 2026-02-05

**Authors:** Zhihui Deng, Jianxin Zhen, Yunan Li, Shuang Liang, Manru Zhang, Siqi Cai, Renhui Jiang, Zhichao Yang, Qiong Yu, Jinyong Wang, Jie Liu

**Affiliations:** 1 Immunogenetics Laboratory, Shenzhen Blood Center, Shenzhen, Guangdong, China; 2 Central Laboratory, Shenzhen Baoan Women’s and Children’s Hospital, Shenzhen, Guangdong, China; 3 Institute of Transfusion Medicine, Shenzhen Blood Center, Shenzhen, Guangdong, China; 4 Department of Blood Transfusion, The Eighth Affiliated Hospital, Sun Yat-Sen University, Shenzhen, Guangdong, China; 5 State Key Laboratory of Organ Regeneration and Reconstruction, Institute of Zoology, Chinese Academy of Sciences, Beijing, China; 6 University of Chinese Academy of Sciences, Beijing, China; 7 Beijing Institute for Stem Cell and Regenerative Medicine, Beijing, China

**Keywords:** ALL, AML, Chinese Southern Han, HLA, KIR, leukemia

## Abstract

**Introduction:**

The killer-cell immunoglobulin-like receptor (*KIR*) gene cluster exhibits complicated diversity in haplotype content, copy-number variation (CNV), and allelic polymorphism. To date, 2,286 distinct *KIR* alleles have been released in the IPD-KIR Database. However, little is known about the impact of high-resolution-level *KIR* allelic polymorphisms on leukemia. Our previous study showed that the *KIR AA* genotype carrying more inhibitory genes conferred differential protection against leukemia in the Chinese Southern Han population. Herein, we hypothesized the impact of *KIR* alleles in the *KIR A* haplotype and cognate human leukocyte antigen (HLA) ligand on leukemia.

**Methods:**

The study cohort included 318 ALL patients, 336 AML patients, and 306 unrelated healthy controls. All the study samples were subject to *HLA-A*, -*B*, and -*C* sequencing-based genotyping (PCR-SBT) and high-resolution *KIR* genotyping for all the seven functional *KIR* genes (*KIR2DL1*, *KIR2DL3*, *KIR2DL4*, *KIR3DL1*, *KIR3DL2*, *KIR3DL3*, and *KIR2DS4*) on the *KIR A* haplotype. *HLA* and *KIR* genotypes were assigned using Assign 4.7.1 software.

**Results:**

In the present study, our high-resolution genetic analysis revealed protective *KIR*–*HLA* interactions in individuals with the *KIR AA* genotype. The strong inhibitory KIR2DL1*00201+*C2* interaction reduced ALL risk (*p* = 0.01), while KIR2DL1*00302 +*C2* (*p* = 0.008), KIR2DL3*00201+*C1* (*p* = 0.03), and KIR3DL1*00501+*Bw4 80I* (*p* = 0.008) interactions protected against AML (*p* < 0.05). However, the functionally weaker inhibitory KIR2DL1*004+*C2* interaction conferred ALL risk (*p* = 0.01) in individuals with the *KIR Bx* genotype. Notably, we found that the allelic polymorphisms of the structure gene *KIR3DL3* were associated with the occurrence of leukemia. KIR3DL3*001 tends to confer protection against AML (8.4% vs. 1.3%, *p* = 0.004, *Pc* = 0.06), whereas KIR3DL3*009 conferred susceptibility to AML (29.3% vs. 47.1%, *p* = 0.001, *Pc =* 0.016). KIR3DL3*001 differs from KIR3DL3*009 by an amino acid substitution of non-charged asparagine (N) to charged histidine (H) in its transmembrane domain, suggesting that this functional variant site KIR3DL3_N300H may play a critical role in the occurrence of leukemia in the Chinese population.

**Conclusion:**

These data suggest that *KIR AA* individuals possess strong inhibitory interactions of *KIR* alleles and *HLA*, arming *KIR AA* ^*+*^ NK cells to meditate stronger alloreactivity and cytotoxicity against leukemia cells with lowered HLA expression. Our findings may provide valuable insights into leukemia pathogenesis and better understanding of the immune mechanisms.

## 1 Introduction

Killer-cell immunoglobulin-like receptors (KIRs) are expressed on the surface of natural killer (NK) cells and a subset of cytotoxic T cells. They regulate effector cell activity through interactions with class I human leukocyte antigen (HLA) ligands on target cells, which play a major role in immune surveillance and elimination of tumor and virus-infected cells (Zuo and Zhao, 2021; Deng et al., 2019; Liu et al., 2021; Putz et al., 2024). To perform this function, NK cells express multiple inhibitory and activating receptors; polymorphic inhibitory receptors educate and license NK cells, enabling them to kill tumor or infected cells exhibiting altered or reduced HLA class I expression (Deng et al., 2019).

The function of KIR on NK cells is dependent on the normal expression of class I HLA ligands on the target cell. Specifically, *HLA-C* alleles form two ligand groups, C1 and C2, based on key amino acids at positions 77 and 80. These groups interact with different inhibitory KIRs: HLA-C1 binds to KIR2DL2/3, while HLA-C2 engages the KIR2DL1 receptor. *HLA-B* alleles are divided into Bw4 and Bw6, but only Bw4 allotypes serve as ligands for KIR3DL1, with binding strength influenced by residue 80 (Ile80 > Thr80). The Bw4 motif also appears in some *HLA-A* alleles (e.g., A*23/24/32), and other *HLA-A* allotypes such as A*03/11 can interact with KIR3DL2.

The *KIR* gene cluster on chromosome 19q13.4 consists of 14 functional genes and 2 pseudogenes, exhibiting complicated diversity in haplotype content, copy-number variation (CNV), and allelic polymorphism. Based on individual gene content, *KIR* genes are classified into two broad haplotypes termed *A* and *B*. The *KIR A* haplotype is defined by a fixed set of nine genes with only *KIR2DS4* as activating genes, whereas *KIR B* haplotypes are more diverse and characterized by the presence of more than one activating *KIR* gene and the absence of *KIR2DS4*. Four framework genes (*KIR3DL3*, *KIR3DP1*, *KIR2DL4*, and *KIR3DL2*) are present on virtually all haplotypes, and every *KIR* haplotype is a combination of a centromeric and a telomeric *KIR* gene motif. Individuals can be grouped into *KIR AA* or *KIR Bx* (including *KIR BB* and *KIR AB*) genotype. To date, 2,286 *KIR* alleles have been recorded in the IPD-KIR Database (Release 2.15) (Robinson et al., 2013).

*KIR* allelic polymorphism is known to dramatically alter the characteristics of the transcribed glycoprotein, influencing the binding affinity, specificity, surface expression, and signaling capacity of the expressed receptor (Bari et al., 2009; Saunders et al., 2016; Nemat-Gorgani et al., 2018; Le Luduec et al., 2019; Wright et al., 2024). The significance of *KIR* allele polymorphisms in clinical transplantation, diseases association, population genetics, and evolution studies has drawn extensive interest recently (Bari et al., 2013; Boudreau et al., 2017; Deng et al., 2021; Guethlein et al., 2021; Palmer et al., 2023).

The existing literature exploring the influence of *KIR* allelic polymorphisms on recipient outcomes after allogeneic hematopoietic stem cell transplantation (HSCT) mainly pointed to donor centromeric (*cen*) *AA KIR2DL* (*2DL1–3*) (Dubreuil et al., 2020), *KIR3DL1* (Boudreau et al., 2017), and *KIR2DL1* allelic/allotype polymorphisms (Bari et al., 2013; Wright et al., 2024). Centromeric (*cen*) *AA* individuals carrying more efficient *KIR2DL* alleles (KIR2DL1*003 and KIR2DL3*001) were associated with better responsiveness and reduced relapse rate in patients with myeloid diseases after T-cell-replete haplo-identical HSCT (Dubreuil et al., 2020). Additionally, KIR3DL1 and HLA-B combinations that were predictive of weak inhibition or noninhibition were associated with significantly lower relapse and overall mortality following allogeneic HSCT in AML patients (Boudreau et al., 2017). Furthermore, *KIR2DL1* alleles with arginine at amino acid position 245 (*KIR2DL1-R* ^*245*^) are functionally stronger than *KIR2DL1* alleles with cysteine at the same position (*KIR2DL1-C* ^*245*^) (Bari et al., 2009). A donor graft containing *KIR2DL1-R* ^*245*^ (KIR2DL1*002/001g, *003, etc.) was associated with better survival and lower cumulative incidence of disease progression in pediatric allogeneic HSCT (Bari et al., 2013). However, the effect of donor *KIR2DL1* allelic diversity on the outcomes of HSCT can be influenced by the conditioning regimen. In a more recent study, donor KIR2DL1*003 with *KIR2DL1-R* ^*245*^ severely reduced disease-free survival and increased 5-year relapse incidence for AML patients receiving reduced intensity conditioning (RIC) and T-cell-depleted (TcD) transplantation, suggesting that KIR2DL1*003-positive donors should be excluded for TcD and RIC transplantation (Wright et al., 2024).

Taken together, the above studies demonstrated that the *KIR* alleles and functional allotypes are apparently associated with the license of NK cells, cancer control, and patient survival after HSCT. However, evaluating the influence of distinct *KIR* allele and HLA ligands on transplantation outcomes is complex as conditioning regimens (conditioning intensity, T-cell-depleted, etc.) may influence the effect of donor *KIR* allelic diversity on HSCT outcomes (Wright et al., 2024). In contrast, the present study focuses on the genetic factors of KIR–HLA interactions mediating protection against or susceptibility to leukemia, without considering the influences of conditioning regimens in transplantation.

Studying *KIR* polymorphism is critical for understanding its anti-leukemic effects and facilitating subsequent application in optimized donor selection in HSCT and immunotherapy (Deng et al., 2019). Based on the low-resolution level diversity of *KIR* genes, numerous studies analyzing potential *KIR/HLA* associations with leukemia have yielded inconsistent results (Babor et al., 2014; de Smith et al., 2014; Vejbaesya et al., 2014; Augusto, 2016; Misra et al., 2016; Shen et al., 2016; Li et al., 2023). The disparate results might be partly due to genetic variation across populations. For instance, de Smith et al. (2014) demonstrated that haplotype *A* was associated with an increased risk of ALL in Hispanics but not in non-Hispanic whites, while homozygosity for *HLA-Bw4* was associated with an increased risk in non-Hispanic whites but not in Hispanics, which demonstrate that the examination of specific well-defined human populations is critical for understanding the role of *KIR* in leukemia control.

AML is a hematological malignancy originating in the bone marrow characterized by a disruption in normal hematopoietic differentiation (Estey and Dohner, 2006), which accounts for approximately 1.3% of all cancer cases and is responsible for approximately 62% of deaths caused by leukemia (Paparo et al., 2025). ALL is a hematological malignancy characterized by the uncontrolled proliferation of immature lymphoid cells, which is an leading cause of cancer-related death in adults (Siegel et al., 2023; Zhang et al., 2023). China had the highest burden of ALL and AML worldwide, accounting for 38,570.94 incidences and 20,612.91 deaths in 2021 (Ni et al., 2025). Our previous study revealed that the frequency of individuals with the *KIR AA* genotype among healthy Chinese Hans is significantly greater than that in other populations representing major world groups (including Amerindian, European, Oceanian, and sub-Saharan African). Moreover, the frequency of *KIR AA* among healthy Chinese Southern Hans (N = 745) is significantly higher than that in adult leukemic patients (N = 836) and pediatric leukemic patients (N = 225), which implies that the *KIR AA* genotype carrying more inhibitory *KIR* genes protects the Chinese Han individuals from leukemia and plays a critical role in human adaptive evolution (Deng et al., 2019). These genetically determined distinctions prompted us to explore the association of allelic polymorphisms in all the seven functional *KIR* genes on the *KIR A* haplotype with acute leukemia occurrence in the Chinese population, aiming at identifying the risk or protective *KIR* alleles or *KIR–HLA* combinations and elucidating potential mechanisms. These findings may provide valuable insights into the pathogenesis of leukemia and facilitate potential application in donor selection for transplantation and NK-cell immunotherapy.

## 2 Materials and methods

### 2.1 Samples from patients and healthy donors

The study cohort included 318 ALL patients, 336 AML patients, and 306 age-matched, unrelated, healthy blood donors with the average age of 31.2, 34.3, and 32.7 years, respectively, as detailed in Supplementary Table S1. The studied adult ALL/AML patients who underwent chemotherapy treatments were recruited from the HSCT program at Shenzhen Blood Center from August 1999 through June 2015. The presence or absence of *KIR* genes had been identified for these patients in our previous study (Deng et al., 2019), but high-resolution *KIR* genotyping was not performed. Individuals with negative results for hepatitis B virus (HBV), hepatitis C virus (HCV), human immunodeficiency virus (HIV), and syphilis were enrolled as healthy controls. Healthy control samples were randomly collected at Shenzhen Blood Center during the period of June 2013 through May 2014. The high-resolution genotypes of the 14 functional *KIRs* had been elucidated for healthy controls, as described previously (Deng et al., 2021). Particularly, the healthy control individuals had no family relationships with the leukemic patients. Written informed consent was obtained from all subjects, and the study was approved by the Ethics Review Board of the Shenzhen Blood Center, Shenzhen, Guangdong, China.

### 2.2 High-resolution *HLA-A*, -*B*, and -*C* genotyping

*HLA-A*, *-B*, and *-C* genotyping was performed using the AlleleSEQR HLA sequencing-based genotyping (PCR-SBT) commercial kit (Atria Genetics, San Francisco, United States). According to the manufacturer’s instructions, exons 2–4 for *HLA-A*, *-B*, and *-C* were sequenced in both directions using an ABI 3730XL DNA sequencer (Applied Biosystems, Foster City, CA, United States). *HLA* genotypes with four-digit resolution were assigned using Assign 4.7.1 software (Conexio Genomics, Fremantle, Australia).

### 2.3 High-resolution-level *KIR* genotyping for AML or ALL patients

We performed high-resolution *KIR* genotyping for all the seven functional *KIR* genes (*KIR2DL1*, *KIR2DL3*, *KIR2DL4*, *KIR3DL1*, *KIR3DL2*, *KIR3DL3*, and *KIR2DS4*) on the *KIR A* haplotype. *KIR* genes that tested positive using PCR-SSP were then subjected to sequencing-based genotyping of all exons for each functional *KIR* gene, as described previously (Deng et al., 2021). *KIR* alleles with at least three-digit resolution were assigned using Assign 4.7.1 software (Conexio Genomics, Fremantle, Australia) with reference to the *KIR* alleles Release 2.13 of the IPD (Robinson et al., 2013). When the sequencing results showed ambiguous allele combinations, we further used group-specific PCR primer pairs to amplify and sequence the target *KIR* alleles separately (Zhang and Deng, 2016). Novel *KIR* alleles were confirmed via TA cloning and sequencing of *KIR* transcripts, as described previously (Deng et al., 2021; Yu et al., 2024).

### 2.4 Statistical analysis

The observed frequencies of *KIR* alleles were calculated by directly counting the number of allele-positive individuals and then dividing it by the total number of studied samples, as described in previous studies (Chen et al., 2022). To compare the frequencies of the identified *KIR* alleles, a chi-squared test was applied using IBM SPSS Statistics 20 software, with a p-value of less than 0.05 indicating statistical significance. Where appropriate, all analyses were corrected using the Bonferroni method (Deng et al., 2019; Chen et al., 2022).

## 3 Results

### 3.1 Characteristics of *KIR* alleles identified in patients and healthy controls

We analyzed all the seven functional *KIR* genes on the *KIR A* haplotype, and a total of 88 distinct *KIR* alleles for healthy controls, 83 for ALL patients, and 89 for AML patients were identified, as summarized in Table 1. Additionally, 28 novel *KIR* alleles were identified and characterized, as summarized in Supplementary Table S2. Ten of these novel alleles having an observed count of ≥3 were identified in leukemic patients.

**TABLE 1:** Number of KIR alleles identified in healthy controls and acute leukemic patients.

KIR gene	Healthy controls (N = 306)		ALL patients (N = 318)		AML patients (N = 336)	
	Number of KIR alleles identified	Number of KIR alleles with a frequency of ≥1%	Number of KIR alleles identified	Number of KIR alleles with a frequency of ≥1%	Number of KIR alleles identified	Number of KIR alleles with a frequency of ≥1%
*2DL1*	10	3	14	5	11	4
*2DL3*	15	4	14	5	15	4
*2DL4*	8	5	6	5	8	5
*2DS4*	7	4	5	4	4	4
*3DL1*	11	6	11	6	12	8
*3DL2*	18	12	16	13	21	12
*3DL3*	19	15	17	13	18	13
Total	88	49	83	51	89	50

The strong inhibitory alleles KIR2DL1*003 and KIR2DL3*001 were common with observed frequencies exceeding 89.3% in healthy controls and ALL/AML patients. KIR3DL1*015, a strong inhibitor upon binding to the Bw4 ligand, is also frequent in the studied subjects. However, the weakly expressed allele KIR3DL1*004, which is present at allele frequencies of 5%–15% in African, European, Oceanic, and South Asian populations (Norman et al., 2007; Deng et al., 2021), is absent in the Chinese Han population. These findings suggest that the Chinese Han population has a strong predisposition to retain a high number of functional inhibitory *KIR* alleles.

In both ALL and AML patients, the telomeric *KIR3DL1* and *KIR3DL2* and centromeric *KIR3DL3* genes, which encode inhibitory NK-cell receptors, each possess two or three high-frequency alleles alongside multiple less frequent alleles. In contrast, the centromeric *KIR2DL1* and *KIR2DL3* genes, which also encode inhibitory receptors, are all dominated by a single high-frequency allele (Supplementary Figure S1 for ALL; Supplementary Figure S2 for AML). This observation agreed well with our previous population study on healthy Chinese Hans (Deng et al., 2021), which indicated that directional selection reduced the sequence diversity of the centromeric *KIR* in the Chinese Han population, whereas the telomeric *KIR* region maintained high diversity caused by balancing selection.

### 3.2 *KIR2DL1* and *KIR2DL3* harbor alleles that tend to confer protection against ALL or AML, while *KIR3DL3* allelic polymorphisms associate with leukemia occurrence

**FIGURE 1:**
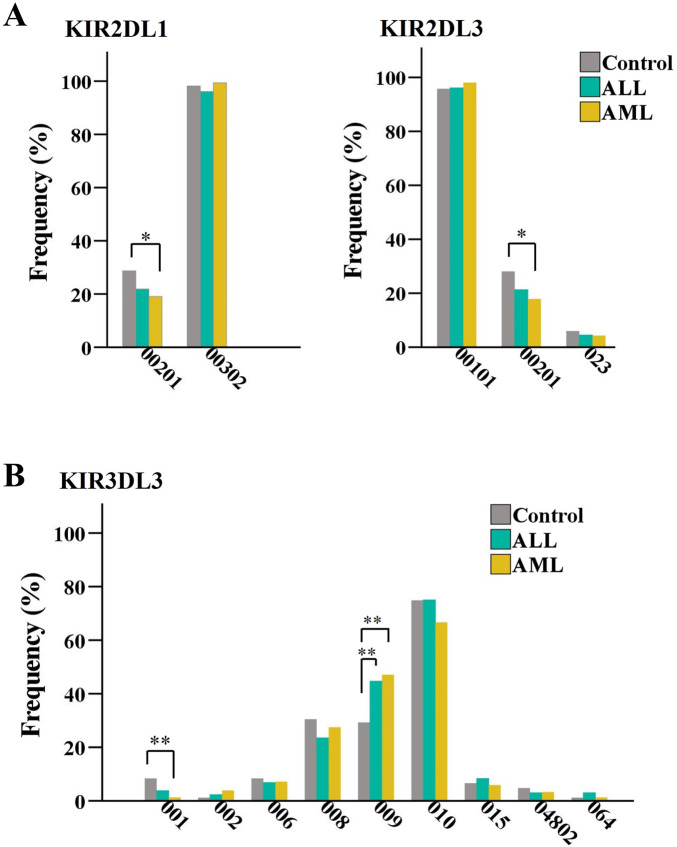
Comparison analysis of the allele frequencies of *KIR2DL1*, *KIR2DL3*, **(A)** and *KIR3DL3* **(B)** genes between healthy controls and ALL or AML patients with the *KIR AA* genotype. *p < 0.05 and **p < 0.01.

To analyze the role of *KIR* alleles in conferring susceptibility or protection against leukemia, healthy controls and leukemic patients were categorized into *KIR AA* and *KIR Bx* groups and then analyzed independently. Comparison of the frequencies of *KIR* alleles between healthy controls and patients with the *KIR AA* genotype (Supplementary Table S3) showed that two centromeric strong inhibitory alleles KIR2DL1*00201 and KIR2DL3*00201 were associated with protection against AML (*p* < 0.05), as shown in Figure 1A. The leukemia-specific novel allele KIR2DL1*069, which was most similar to KIR2DL1*00302 with two amino acid substitutions (308Ile > Thr and 309Ile > Arg) in cytoplasmic regions, showed susceptibility to AML (*p* = 0.04). However, the significant differences in these alleles were lost after *p-*value correction (Table 2).

**TABLE 2:** Significant differences in the observed frequencies of *KIR* alleles between healthy controls and ALL/AML patient groups with the *KIR AA* genotype.

KIR allele	Control (N = 167)	ALL patients (N = 155)				AML patients (N = 162)			
	n (%)	n (%)	OR (95% CI)	*P*	*P*_*c*_	n (%)	OR (95% CI)	*P*	*P*_*c*_
2DL1*00201	48 (28.7)	34 (21.9)	0.70 (0.42–1.16)	0.16	​	31 (19.1)	0.59 (0.35–0.98)	**0.04**	0.41
2DL1*069	0 (0)	5(3.2)	0.97 (0.94–1.00)	0.06	​	6 (3.7)	0.96 (0.93–0.99)	**0.04**	0.36
2DL3*00201	47 (28.1)	33(21.3)	0.69 (0.41–1.15)	0.16	​	29 (17.9)	0.56 (0.33–0.94)	**0.03**	0.50
3DL3*001	14 (8.4)	5(3.8)	0.43 (0.15–1.23)	0.11	​	2 (1.3)	0.14 (0.03–0.65)	**0.004**	0.06
3DL3*009	49 (29.3)	59(44.7)	1.95 (1.21–3.14)	**0.01**	0.08	72 (47.1)	2.14 (1.35–3.39)	**0.001**	**0.016**

The p-values less than 0.05 indicating statistical significance were highlighted in bold.

For the first time, we found that the allelic polymorphism of the structure gene *KIR3DL3* was associated with leukemia occurrence (Table 2; Figure 1B). Compared to healthy controls, KIR3DL3*001 showed a decreased frequency in AML groups (8.4% vs. 1.3%, *p* = 0.004), suggesting that KIR3DL3*001 tends to confer protection against AML, though the difference did not reach statistical significance (*Pc* = 0.06). In contrast, the frequency of KIR3DL3*009 significantly increased in the AML (29.3% vs. 47.1%, *p* = 0.001) and ALL (29.3% vs. 44.7%, *p* = 0.01) groups, indicating that KIR3DL3*009 conferred susceptibility to AML/ALL (AML: *Pc* = 0.016; ALL: *Pc* = 0.08). KIR3DL3*001 differs from KIR3DL3*009 by a single missense mutation at Codon 300 AAC>CAC in exon 7, which results in an amino acid substitution of non-charged asparagine (N) to charged histidine (H) in its transmembrane domain. Our results indicate that this functional variant site KIR3DL3_N300H plays a critical role in the occurrence of leukemia in the Chinese population.

As for the healthy controls and leukemic patients with the *KIR Bx* genotype, no *KIR* alleles showed significant difference (Supplementary Table S4).

### 3.3 *KIR3DL3_N300* and *KIR3DL3_H300* showed an association with the occurrence of AML in the Chinese population

**FIGURE 2:**
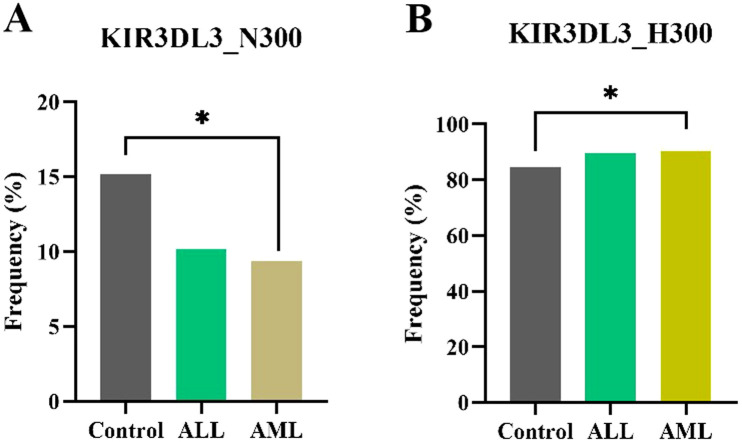
Association of the *KIR3DL3_300* allotypes with the occurrence of leukemia. Compared to healthy controls, **(A)** *KIR3DL3_N300* showed a decreased frequency in AML (15.2% vs. 9.4%, *p = 0.03) and **(B)** *KIR3DL3_H300* showed an increased frequency in AML (84.7% vs. 90.5%, *p = 0.03).

Based on the residue in position 300, *KIR3DL3* alleles were grouped as *KIR3DL3_N300* (KIR3DL3*001, *006, etc.), *KIR3DL3_H300* (KIR3DL3*002, *008, *009, etc.), and *KIR3DL3_Y300* (KIR3DL3*003, *004, etc.). Compared to healthy controls, *KIR3DL3_N300* showed a decreased frequency in AML (15.2% vs. 9.4%, *p* = 0.03), whereas *KIR3DL3_H300* showed an increased frequency in AML (84.7% vs. 90.5%, *p* = 0.03), as shown in Figures 2A,B and Supplementary Table S5. Thus, the functional allotype analysis of *KIR3DL3_300* agreed well with the data for the association of *KIR3DL3* allelic polymorphisms with AML or ALL. Notably, *KIR3DL3* alleles with *KIR3DL3_Y300*, which is common in Caucasians, African Americans, and Māori populations with a frequency of 33.4%, 33.5%, and 32.7%, respectively (Hou et al., 2011; Vierra-Green et al., 2012; Nemat-Gorgani et al., 2014), is absent in Chinese Hans.

Other allotypes including *KIR2DL1-P114*/*KIR2DL1-L114* (Hilton et al., 2015) and *KIR2DL1-R245*/*KIR2DL1-C245* (Bari et al., 2011; Bari et al., 2013; Hilton et al., 2015) showed no significant difference in the frequencies of these allotypes between healthy controls and ALL/AML patients with the *KIR AA* genotype. Neither the KIR3DL1-H nor the KIR3DL1-L allotype (Gardiner et al., 2001; Boudreau et al., 2014) was associated with the risk of ALL/AML (Supplementary Table S5 *).*

### 3.4 HLA-C2 ligand was associated with protection against AML in individuals with the *KIR AA* genotype

To evaluate the role of HLA ligands in conferring susceptibility or protective effects, we compared the observed frequencies of HLA ligands between the healthy controls and ALL/AML patients with the *KIR AA* genotype; the strong inhibitory HLA-C2 ligand was associated with a protective effect against AML (31.1% vs. 17.9%, *P =* 0.005), and the difference remained statistically significant after *p-*value correction using the Bonferroni method (*Pc* = 0.01), as shown in Supplementary Table S6.

For the healthy controls and leukemic patients with the *KIR Bx* genotype, no HLA ligands showed significant difference.

### 3.5 *KIR AA* individuals possessing strong inhibitory *KIR* alleles in the presence of cognate ligands show protective effects against ALL or AML, while *KIR Bx* individuals possessing the functionally weaker KIR2DL1*004+HLA-C2 interaction confer susceptibility to ALL

**FIGURE 3:**
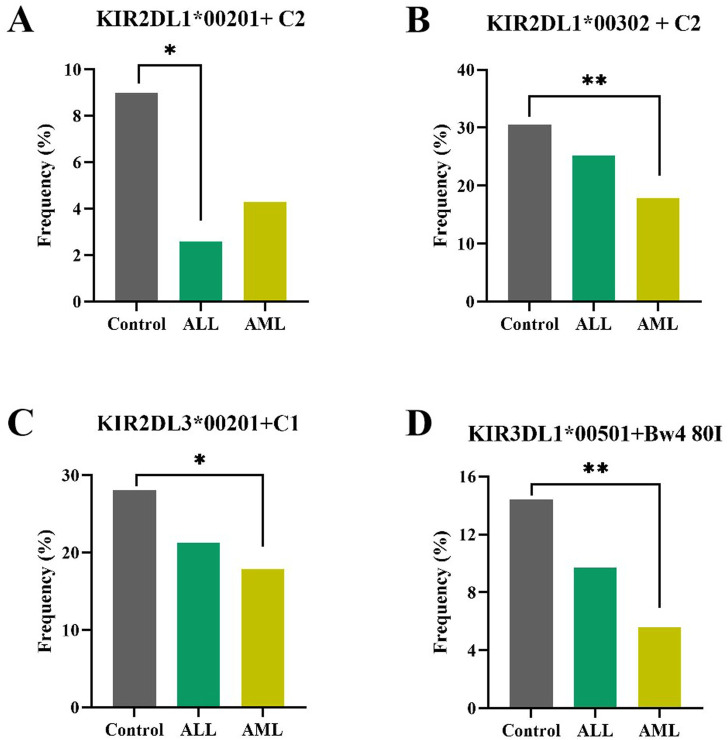
Specific *KIR*–*HLA* interactions were significantly associated with protective effects against leukemia in individuals with the *KIR AA* genotype. (A) The KIR2DL1*00201+*C2* interaction showed a significantly decreased frequency in ALL patients compared to healthy controls (2.6% vs. 9.0%, *p = 0.01). **(B)** The KIR2DL1*00302+*C2* interaction showed decreased frequencies in AML patients compared to healthy controls (17.9% vs. 30.5%, **p = 0.008). **(C)** The KIR2DL3*00201+C1 interaction showed decreased frequencies in AML patients compared to healthy controls (17.9% vs. 28.1%, *p = 0.03). **(D)** The KIR3DL1*00501+*Bw4 80I* interaction showed decreased frequencies in AML patients compared to healthy controls (5.6% vs. 14.4%, **p = 0.008). *p < 0.05 and **p < 0.01.

In individuals with the *KIR AA* genotype, specific *KIR*–*HLA* interactions were found to be significantly associated with protective effects against leukemia (Table 3; Supplementary Table S7). As shown in Figure 3A, the KIR2DL1*00201+*C2* interaction showed a significantly decreased frequency in ALL patients compared to healthy controls (2.6% vs. 9.0%, *p* = 0.01). Similarly, KIR2DL1*00302+*C2*, KIR2DL3*00201+*C1*, and KIR3DL1*00501+*Bw4 80I* interactions all showed decreased frequencies in AML patients compared to healthy controls (2DL1*00302+*C2*: 17.9% vs. 30.5%, *p* = 0.008; 2DL3*00201+C1: 17.9% vs. 28.1%, *p* = 0.03; 3DL1*00501+*Bw4 80I*: 5.6% vs. 14.4%, *p* = 0.008), as shown in Figures 3B–D. These results suggested that individuals with the *KIR AA* genotype possessing strong inhibitory *KIR* alleles (2DL1*00201, 2DL1*00302, 2DL3*00201, and 3DL1*00501) in the presence of cognate ligands conferred protection against ALL or AML in Chinese Hans.

**TABLE 3:** Significant differences in the observed frequencies of KIR–HLA interactions between healthy controls and ALL/AML patient groups with the *KIR AA* genotype.

*KIR* genotype of study subjects	*KIR*–*HLA* ligand	Control	ALL			AML		
		n (%)	n (%)	OR (95% CI)	*P*	n (%)	OR (95% CI)	*P*
*KIR AA*	2DL1*00201+C2	15 (9.0)	4 (2.6)	0.27 (0.09-0.83)	**0.01**	7 (4.3)	0.46 (0.18-1.15)	0.09
​	2DL1*00302+C2	51 (30.5)	39 (25.2)	0.76 (0.47-1.25)	0.28	29 (17.9)	0.50 (0.30-0.83)	**0.008**
​	2DL3*00201+C1	47 (28.1)	33 (21.3)	0.69 (0.41-1.15)	0.16	29 (17.9)	0.56 (0.33-0.94)	**0.03**
​	3DL1*00501+Bw4 80I	24 (14.4)	15 (9.7)	0.64 (0.32-1.27)	0.2	9 (5.6)	0.35 (0.16-0.78)	**0.008**
*KIR Bx*	2DL1*004+*C2*	0 (0)	9 (5.5)	0.94 (0.91-0.98)	**0.01**	6 (3.4)	0.97 (0.94-0.99)	0.07

In table 3, the number of individuals with the *KIR AA* genotype in the healthy control, ALL, and AML groups is 167, 155, and 162, respectively. Individuals with the *KIR Bx* genotype in the healthy control, ALL, and AML groups is 139, 163, and 174, respectively. The p-values less than 0.05 indicating statistical significance were highlighted in bold.

In contrast, analysis of the healthy controls and leukemic patients with the *KIR Bx* genotype showed that the functionally weaker KIR2DL1*004+HLA-C2 interaction conferred susceptibility to ALL (*p* = 0.01, Table 3). No other KIR–HLA interaction was found to be associated with the occurrence of ALL or AML (Supplementary Table S8).

## 4 Discussion

Our previous study revealed that the *KIR AA* genotype carrying more inhibitory *KIR* genes confers protective effects against leukemia in the Chinese Han population (Deng et al., 2019). This observation prompted us to explore the association of allelic polymorphisms in all the seven functional *KIR* genes on the *KIR A* haplotype with acute leukemia. As the allelic diversity of the *KIR B*-haplotype-specific genes (*2DL2*, *2DL5*, *2DS1*, *2DS2*, *2DS3*, *2DS5*, and *3DS1*) is limited (Deng et al., 2021) and the frequencies of the *KIR B*-haplotype-specific genes in ALL/AML patients showed no statistically significant difference in the healthy controls (Deng et al., 2019), we did not analyze the association of *KIR B*-haplotype-specific alleles with the occurrence of AML/ALL.

In the present study, we found that individuals with the *KIR AA* genotype possessed strong inhibitory *KIR* alleles for *2DL1*, *2DL3*, and *3DL1* in association with cognate ligands that are protective against leukemia in the Chinese population. High-resolution genetic analysis indicated that the strong inhibitory interaction KIR2DL1*00201+*C2* conferred protective effects against ALL, while KIR2DL1*00302+*C2*, KIR2DL3*00201+*C1*, and KIR3DL1*00501+*Bw4 80I* interactions exerted protective effects against AML. However, the functionally weaker KIR2DL1*004+HLA-C2 interaction was associated with ALL risk (*p* = 0.01) among individuals with the *KIR Bx* genotype.

A previous study showed that when both the donor and host expressed HLA-C1/HLA-C2/HLA-Bw4 ligands, NK cells expressing three inhibitory receptors (2DL1/2DL3/3DL1) exhibited maximum responsiveness against K562 cells and contributed to the lowest relapse rates in patients with AML and myelodysplastic syndrome following haplo-HSCT in the Chinese population (Zhao et al., 2019). *KIR3DL1* alleles showed differential expression level and inhibitory capacity, and KIR3DL1*005 combines the low frequency and level of expression with strong inhibitory function (Yawata et al., 2006). Interestingly, KIR3DL1*005 and its associated haplotypes are associated with superior tyrosine kinase inhibitor (TKI) therapeutic effects, and the combinations of these *KIR* and *HLA* alleles may correlate with potent NK-cell immunity against CML (Ureshino et al., 2018). These studies further substantiated the reliability of our findings (Ureshino et al., 2018; Zhao et al., 2019). Moreover, numerous studies demonstrated that strongly inhibiting KIR receptors enable education and maturation of NK cells with strong effector functions (Yawata et al., 2006; Kim et al., 2008; Brodin et al., 2009), and higher numbers of inhibitory KIR ligands promote increased numbers of circulating NK cells, stronger killing capacity, and greater NK-cell repertoire diversity (Yawata et al., 2006; Brodin et al., 2009; Deng et al., 2021). These reports may partly explain why strong inhibitory *KIR* alleles for *KIR2DL1*, *KIR2DL3*, and *KIR3DL1* in the presence of cognate ligands confer protective effects against leukemia in the Chinese population.

In the context of tumors and chronic infections, NK-cell exhaustion is characterized by the downregulated expression of activating receptors (e.g., NKG2D and FCGR3A), upregulated expression of inhibitory receptors (e.g., KLRC1, PD-1, TIGIT, and Tim-3), decreased production of effector cytokines (e.g., IFNγ and GZMB), and impaired cytolytic activity (Bi and Tian, 2017; Zhang et al., 2018; Jin et al., 2024). Previous reports demonstrated that the absence of inhibitory KIR–HLA interactions is associated with the induction of NK-cell exhaustion (Ardolino et al., 2014; Seo et al., 2017). We speculated that NK cells with the *KIR AA* genotype, which carries more inhibitory KIRs paired with cognate HLA ligands, may exhibit enhanced protection against exhaustion induced by prolonged activation. However, this hypothesis necessitates further experimental validation to confirm its validity.

*KIR2DL1* alleles with *KIR2DL1-R* ^*245*^ (KIR2DL1*002/001g, *003, etc.) are functionally stronger than *KIR2DL1* alleles with *KIR2DL1-C245* (KIR2DL1*004, *007, etc.) (Bari et al., 2009). Both KIR2DL1*002 ^+^ NK cells and KIR2DL1*003 ^+^ NK cells have a stronger degranulation compared to KIR2DL1*004 ^+^ NK cells in *C2* ^+^ donors (Dubreuil et al., 2020). The common KIR2DL1*003, less common KIR2DL1*002, and *Cen-B*-associated KIR2DL1*004 are the three dominant *KIR2DL1* alleles identified in the Chinese population. In the presence of the HLA-C2 ligand, the functionally stronger inhibitory KIR2DL1*002 and *003 alleles carrying *KIR2DL1-R* ^*245*^ exhibit protective effects against ALL or AML in Chinese individuals with the *KIR AA* genotype, while the functionally weaker inhibitory allele KIR2DL1*004 having *KIR2DL1-C* ^*245*^ confers ALL risk in individuals with the *KIR Bx* genotype. Our findings are consistent with previous studies (Bari et al., 2013), which reported that patients who received a donor graft containing *KIR2DL1-R* ^*245*^ had lower cumulative incidence of disease progression and better survival in allogeneic HSCT.

Among the 14 functional *KIR* genes, the framework gene *KIR3DL3* is highly polymorphic with 235 alleles, which has been shaped by natural selection (Leaton et al., 2019). However, *KIR3DL3* expression is inhibited by methylation of the promoter and is restored to the cell surface at similar levels to other *KIR* genes upon demethylation (Trompeter et al., 2005; Trundley et al., 2006). In the NK cells extracted from the blood of healthy human adults, KIR3DL3 expression is detected at a low level (Torkar et al., 1998; Long et al., 2001), but it is more common in the lungs and digestive tract (Palmer et al., 2023). KIR3DL3 interacts with the B7 family protein HERV-H LTR-associating 2 (HHLA2), which mediates inhibition in T cells and NK cells and is implicated for immune checkpoint targeting (Verschueren et al., 2020; Wojtowicz et al., 2020; Bhatt et al., 2021). However, little is known about the biological function of the KIR3DL3 molecule and the significance of its allelic polymorphisms. Our study, for the first time, found an association of *KIR3DL3* allelic diversity with leukemia. KIR3DL3*001 tended to confer protection against AML (*P* = 0.004, *Pc* = 0.06). In contrast, KIR3DL3*009 conferred susceptibility to AML (*Pc* = 0.016). KIR3DL3*001 differs from KIR3DL3*009 by a single missense mutation at Codon 300 AAC>CAC, resulting in an amino acid substitution of non-charged asparagine (N) to charged histidine (H) in its transmembrane domain, suggesting that the functional variant KIR3DL3_N300H plays a critical role in protection against or susceptibility to ALL or AML in Chinese Hans. The further comparative analysis for *KIR3DL3_300* allotypes also indicated that *KIR3DL3_N300* alleles conferred protection against AML, whereas *KIR3DL3_H300* alleles were associated with an increased risk of AML. Our findings may provide new insights into the role of *KIR3DL3* polymorphisms in leukemia pathogenesis.

One limitation of this study is that the key aspects of the *KIR3DL3* mechanism and the functional impact of its polymorphisms on leukemia remain to be elucidated. For leukemic patients, we failed to determine the expression of KIR3DL3 on NK cells and HHLA2 expression on leukemic blast, though HHLA2 is expressed and upregulated in tumors of the lung, gastrointestinal tract, kidney, and liver (Chen et al., 2019; Jing et al., 2019). Our future work will include the construction of NK cells with homozygous KIR3DL3*001 ^+^ or KIR3DL3*009 ^+^ and NK-cell cytotoxicity assay against HHLA2-positive target cells. Another limitation is that the relatively low sample numbers we obtained for the NK-cell cytotoxicity tests did not allow for a thorough analysis and validation of interactions between *KIR* allele and cognate ligand that conferred protection or susceptibility to AML/ALL among the randomly selected NK-cell donors and target cells. Such experiments require pre-genotyping of a large number of NK-cell donors.

In summary, the present study investigated the *KIR* allelic polymorphisms of all the seven functional *KIR* genes on the *KIR A* haplotype in Chinese AML/ALL patients and healthy controls. We found that individuals with the *KIR AA* genotype possess strong inhibitory *KIR* alleles in association with cognate ligands that are protective against acute leukemia. Notably, the allelic polymorphisms of the structure gene *KIR3DL3* were associated with leukemia occurrence. Our findings may provide valuable insights into leukemia pathogenesis and better understanding of the immune mechanisms.

## Data Availability

The original contributions presented in the study are included in the article/Supplementary Material, further inquiries can be directed to the corresponding authors.

## Supplementary material

SUPPLEMENTARY TABLE 1 Supplementary Tables S1-S8.

SUPPLEMENTARY DATA SHEET 1 Supplementary Figures S1-S2.
