# Supplementary material for: KIR AA individuals possess strong inhibitory KIR alleles alongside HLA ligands that are protective against leukemia in the Chinese population
Source: Front Genet. 2026 Feb 5;16:1745482. doi: 10.3389/fgene.2025.1745482 (PMC12916065; doi:10.3389/fgene.2025.1745482)
Supplement: Supplementary file 1 [file DataSheet1.doc]

**
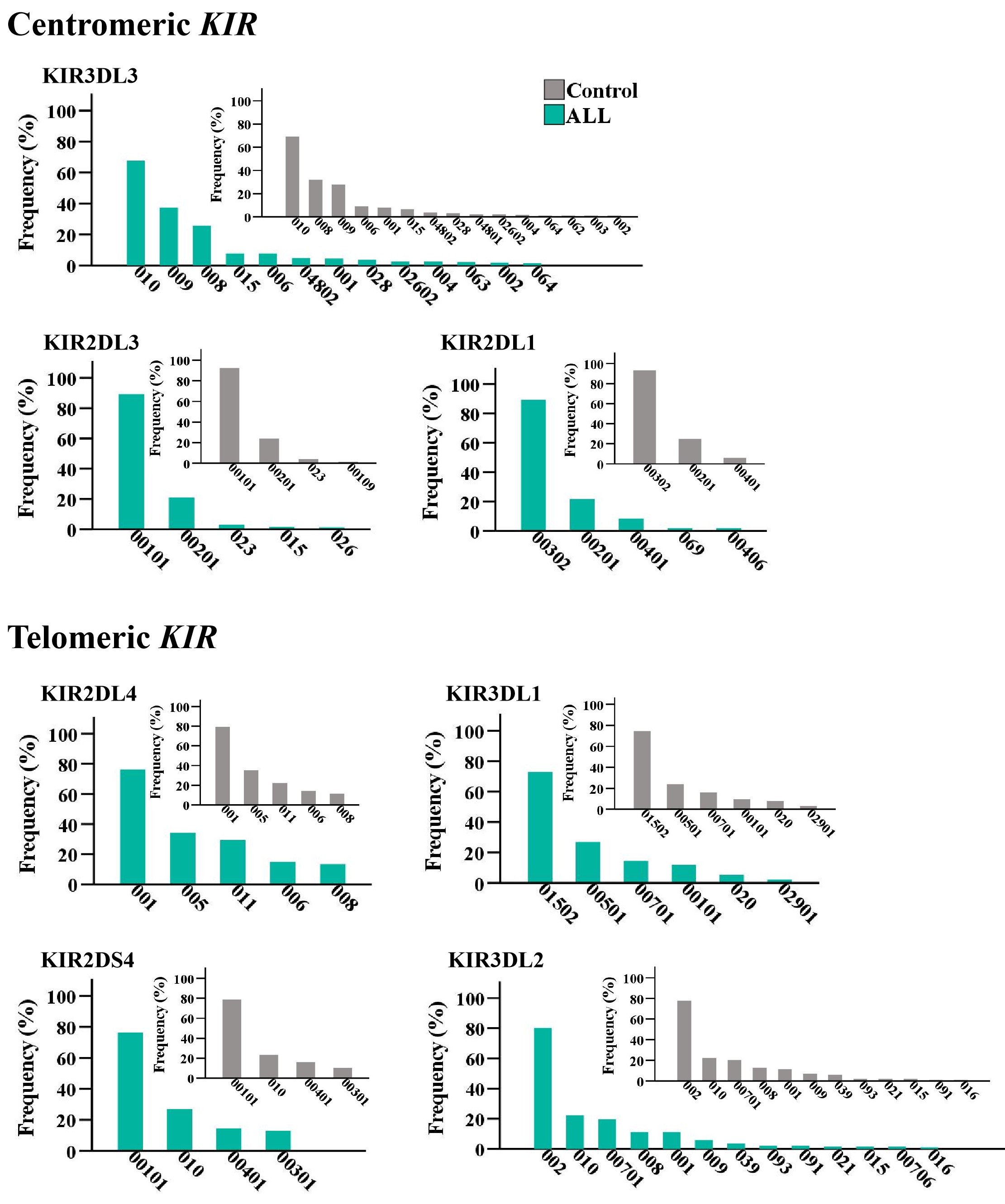
**

**Figure S1 Frequency distribution of *KIR* alleles between healthy controls and ALL patients**. Only *KIR* alleles with frequencies greater than 1% are shown.


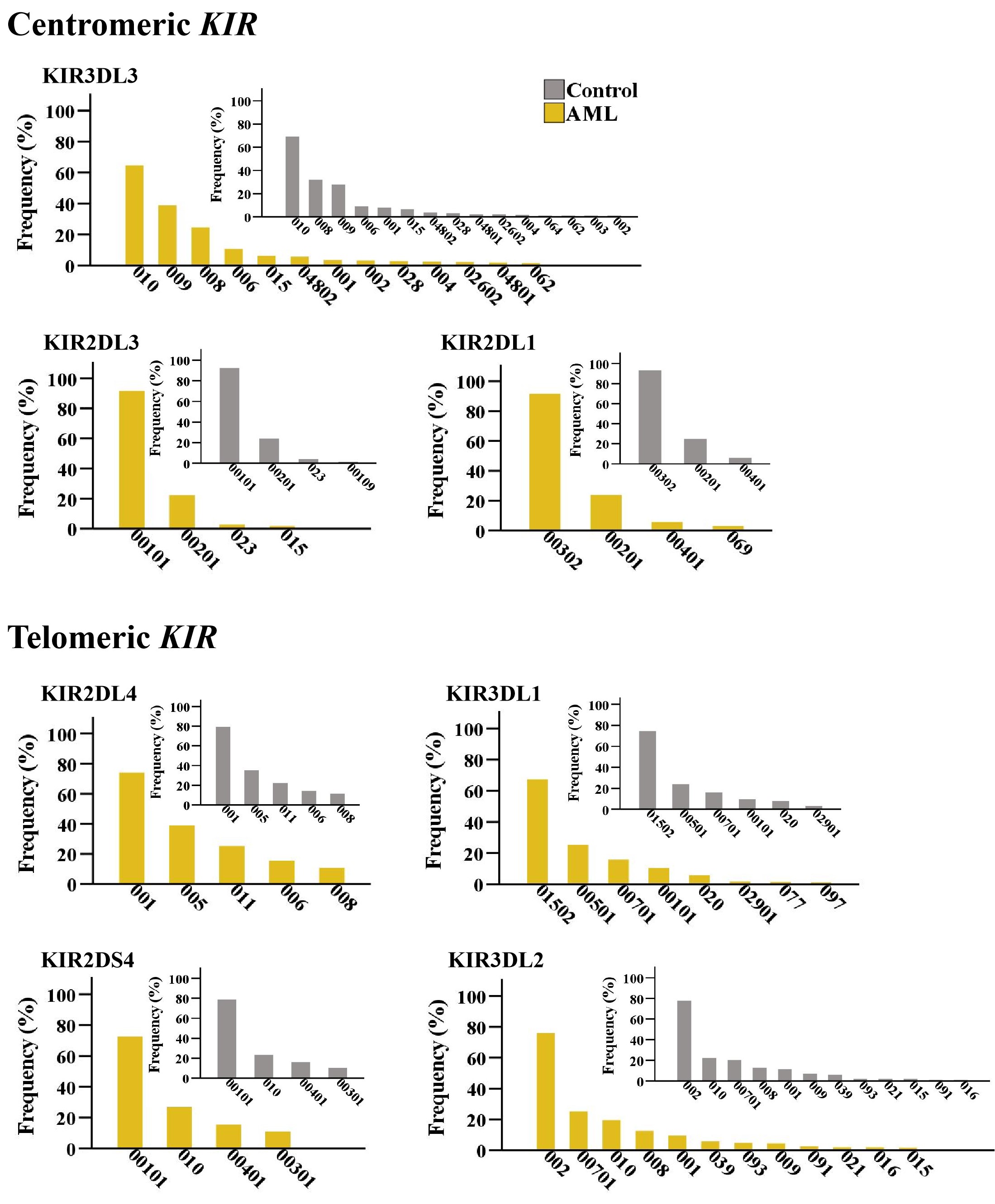


**Figure S2 Frequency distribution of *KIR* alleles between healthy controls and AML patients.** Only *KIR* alleles with frequencies greater than 1% are shown.
