# Supplementary material for: KIR AA individuals possess strong inhibitory KIR alleles alongside HLA ligands that are protective against leukemia in the Chinese population
Source: Front Genet. 2026 Feb 5;16:1745482. doi: 10.3389/fgene.2025.1745482 (PMC12916065; doi:10.3389/fgene.2025.1745482)
Supplement: Supplementary file 2 [file Table1.doc]

**Supplementary Tables**

**Table S1** Characteristics of healthy controls and acute leukemia patients of Chinese Southern Han.

|  | Controls | ALL | AML |
| --- | --- | --- | --- |
| N | 306 | 318 | 336 |
| Female | 14.4% | 38.7% | 46.5% |
| Male | 85.6% | 61.3% | 53.5% |
| Mean age | 32.7 | 31.2 | 34.3 |
| Range | 18-55 | 18-58 | 18-66 |

**Table S2** Characterization of 28 novel alleles identified in AML or ALL patients

| ***KIR*** | **Novel**  **allele name** | **GenBank**  **Accession** | **IPD-KIR**  **Accession** | **Most similar allele** | **Nucleotide change (position in full CDS)** | **Exon** | **Substitution**  **(Codon Number)** | **Amino acid substitution** | **Number**  **observed** | **ref** |
| --- | --- | --- | --- | --- | --- | --- | --- | --- | --- | --- |
| *2DL1* | *2DL1*00308* | MZ361341 | IWS40002700 | *2DL1*00302* | 825 G>A | 8 | 254 GCG>GCA | no change | 1 |  |
|  | *2DL1*00309* | MZ673561 | IWS40002716 | *2DL1*00302* | 102 C>T | 4 | 13 CAC>CAT | no change | 1 |  |
|  | *2DL1*00406* | MZ673558 | IWS40002710 | *2DL1*00401* | 144 A>G | 4 | 27 CAA>CAG | no change | 10 |  |
|  | *2DL1*069* | MZ488844 | IWS40002702 | *2DL1*00302* | 986 T>C | 9 | 308 ATC>ACC | Ile>Thr | 18 |  |
|  |  |  |  |  | 989 T>G | 9 | 309 ATC>AGA | Ile>Arg |  |  |
|  |  |  |  |  | 990 C>A | 9 |  |  |  |  |
|  | *2DL1*070* | MZ673559 | IWS40002712 | *2DL1*00401* | 110 G>C | 4 | 16 CGC>CCC | Arg>Pro | 3 |  |
|  | *2DL1*071* | MZ673560 | IWS40002714 | *2DL1*00302* | 668 T>C | 6 | 209 TCA>CCA | Ser>Pro | 2 |  |
|  | *2DL1*072* | MZ673562 | IWS40002718 | *2DL1*00302* | 415 G>T | 5 | 118 GTT>TTT | Val>Phe | 1 |  |
|  | *2DL1*073* | MZ673563 | IWS40002720 | *2DL1*00201* | 266 G>A | 4 | 68 CGC>CAC | Arg>His | 1 |  |
|  | *2DL1*074* | MZ673564 | IWS40002722 | *2DL1*00302* | 701 C>A | 6 | 213 CCA>CAA | Pro>Gln | 1 |  |
| *2DL3* | *2DL3*00111* | MZ361338 | IWS40002694 | *2DL3*00101* | 564 C>G | 5 | 167 GGC>GGG | no change | 3 |  |
|  | *2DL3*00112* | MZ361340 | IWS40002698 | *2DL3*00101* | 594 C>T | 5 | 177 TGC>TGT | no change | 3 |  |
|  | *2DL3*030* | KF766502 | IWS40001875 | *2DL3*00101* | 201 G>C | 3 | 46 AAG>AAC | Lys>Asn | 2 |  |
|  | *2DL3*032* | KF849248 | IWS40001879 | *2DL3*00101* | 754 G>C | 5 | 231 GTG>CTG | Val>Leu | 1 |  |
|  | *2DL3*037* | MZ361339 | IWS40002696 | *2DL3*00101* | 994 G>C | 9 | 311 GTG>CTG | Val>Leu | 1 |  |
| *2DL4* | *2DL4*00106* | KY974371 | IWS40002350 | *2DL4*00102* | 984 C>T | 9 | 305 AGC>AGT | no change | 1 |  |
|  | *2DL4*00108* | MZ334488 | IWS40002692 | *2DL4*00102* | 624 C>T | 5 | 185 GAC>GAT | no change | 2 |  |
|  | *2DL4*039* | KY974369 | IWS40002346 | *2DL4*00602* | 412 G>A | 5 | 115 GCA>ACA | Ala>Thr | 1 |  |
|  | *2DL4*040* | KY974370 | IWS40002348 | *2DL4*00102* | 524 A>G | 5 | 152 AAT>AGT | Asn>Ser | 1 |  |
| *3DL1* | *3DL1*00702* | MW560122 | IWS40002676 | *3DL1*00701* | 732 T>C | 5 | 223 TGT>TGC | no change | 1 |  |
|  | *3DL1*00703* | MZ334486 | IWS40002690 | *3DL1*00701* | 162 G>A | 3 | 33 AGG>AGA | no change | 1 |  |
|  | *3DL1*01507* | MZ334487 | IWS40002688 | *3DL1*01502* | 1008 C>A | 7 | 315 CCC>CCA | no change | 1 |  |
|  | *3DL1*01508* | MZ673556 | IWS40002706 | *3DL1*01502* | 519 T>C | 4 | 152 TCG>TTG | no change | 1 |  |
|  | *3DL1*120* | MZ673555 | IWS40002704 | *3DL1*00501* | 5 C>T | 1 | -20 TCG>TTG | Ser>Leu | 1 |  |
|  | *3DL1*121* | MZ673557 | IWS40002708 | *3DL1*00101* | 337 G>C | 3 | 92 GTG>CTG | Val>Leu | 4 |  |
| *3DL3* | *3DL3*01503* | OP204419 | IWS40002760 | *3DL3*01501* | 969 C>T | 7 | 302 CAC>CAT | no change | 12 |  |
|  | *3DL3*116* | OP204420 | IWS40002762 | *3DL3*00902* | 724 T>G | 5 | 221 TTG>GTG | Leu>Val | 6 |  |
|  | *3DL3*117* | OP204421 | IWS40002764 | *3DL3*01002* | 336 T>C | 3 | 91 CCT>CCC | no change | 3 |  |
|  |  |  |  |  | 337 G>C | 3 | 92 GTG>CTG | Val>Leu |  |  |
|  | *3DL3*118* | OP204422 | IWS40002766 | *3DL3*01002* | 502 A>G | 4 | 147 ATT>GTT | Ile>Val | 14 |  |

**Table S3**  Comparison analysis of the observed frequencies for *KIR* alleles between healthy controls and ALL/AML patient groups having the *KIR AA* genotype.

| Allele | Control (N=167) |  | ALL (N=155) | | | |  | AML (N=162) | | | |
| --- | --- | --- | --- | --- | --- | --- | --- | --- | --- | --- | --- |
|  | n (%) |  | n (%) | OR (95% CI) | *P* | *P_c_* |  | n (%) | OR (95% CI) | *P* | *P_c_* |
| *2DL1*001* | 0(0) |  | 1(0.6) | 0.99(0.98-1.01) | 0.48 |  |  | 0(0) | - | - |  |
| *2DL1*00201* | 48(28.7) |  | 34(21.9) | 0.70(0.42-1.16) | 0.16 |  |  | 31(19.1) | 0.59(0.35-0.98) | **0.04** | 0.41 |
| *2DL1*00302* | 164(98.2) |  | 149(96.1) | 0.45(0.11-1.85) | 0.43 |  |  | 161(99.4) | 2.95(0.30-28.61) | 0.64 |  |
| *2DL1*00304* | 0(0) |  | 0(0) | - | - |  |  | 1(0.6) | 0.99(0.98-1.01) | 0.49 |  |
| *2DL1*00308* | 0(0) |  | 0(0) | - | - |  |  | 1(0.6) | 0.99(0.98-1.01) | 0.49 |  |
| *2DL1*00309* | 0(0) |  | 0(0) | - | - |  |  | 1(0.6) | 0.99(0.98-1.01) | 0.49 |  |
| *2DL1*00401* | 0(0) |  | 0(0) | - | - |  |  | 1(0.6) | 0.99(0.98-1.01) | 0.49 |  |
| *2DL1*030* | 1(0.6) |  | 0(0) | 1.01(0.99-1.02) | 1.00 |  |  | 0(0) | 1.01(0.99-1.02) | 1.00 |  |
| *2DL1*033* | 1(0.6) |  | 0(0) | 1.01(0.99-1.02) | 1.00 |  |  | 0(0) | 1.01(0.99-1.02) | 1.00 |  |
| *2DL1*034* | 1(0.6) |  | 1(0.6) | 1.08(0.07-17.38) | 1.00 |  |  | 0(0) | 1.01(0.99-1.02) | 1.00 |  |
| *2DL1*069* | 0(0) |  | 5(3.2) | 0.97(0.94-1.00) | 0.06 |  |  | 6(3.7) | 0.96(0.93-0.99) | **0.04** | 0.36 |
| *2DL1*072* | 0(0) |  | 1(0.6) | 0.99(0.98-1.01) | 0.48 |  |  | 0(0) | - | - |  |
| *2DL1*073* | 0(0) |  | 1(0.6) | 0.99(0.98-1.01) | 0.48 |  |  | 0(0) | - | - |  |
| *2DL1*074* | 0(0) |  | 1(0.6) | 0.99(0.98-1.01) | 0.48 |  |  | 0(0) | - | - |  |
| *2DL3*00101* | 160(95.8) |  | 149(96.1) | 1.09(0.36-3.31) | 0.88 |  |  | 159(98.1) | 2.32(0.59-9.13) | 0.36 |  |
| *2DL3*00103* | 0(0) |  | 1(0.6) | 0.99(0.98-1.01) | 0.48 |  |  | 1(0.6) | 0.99(0.98-1.01) | 0.49 |  |
| *2DL3*00108* | 0(0) |  | 0(0) | - | - |  |  | 2(1.2) | 0.99(0.97-1.00) | 0.24 |  |
| *2DL3*00109* | 1(0.6) |  | 1(0.6) | 1.08(0.07-17.38) | 1.00 |  |  | 0(0) | 1.01(0.99-1.02) | 1.00 |  |
| *2DL3*00110* | 0(0) |  | 0(0) | - | - |  |  | 1(0.6) | 0.99(0.98-1.01) | 0.49 |  |
| *2DL3*00111* | 0(0) |  | 1(0.6) | 0.99(0.98-1.01) | 0.48 |  |  | 0(0) | - | - |  |
| *2DL3*00112* | 0(0) |  | 0(0) | - | - |  |  | 1(0.6) | 0.99(0.98-1.01) | 0.49 |  |
| *2DL3*00201* | 47(28.1) |  | 33(21.3) | 0.69(0.41-1.15) | 0.16 |  |  | 29(17.9) | 0.56(0.33-0.94) | **0.03** | 0.50 |
| *2DL3*015* | 1(0.6) |  | 2(1.3) | 2.17(0.19-24.17) | 0.95 |  |  | 2(1.2) | 2.08(0.19-23.11) | 0.98 |  |
| *2DL3*019* | 1(0.6) |  | 1(0.6) | 1.08(0.07-17.38) | 1.00 |  |  | 2(1.2) | 2.08(0.19-23.11) | 0.98 |  |
| *2DL3*021* | 1(0.6) |  | 0(0) | 1.01(0.99-1.02) | 1.00 |  |  | 0(0) | 1.01(0.99-1.02) | 1.00 |  |
| *2DL3*022* | 2(1.2) |  | 0(0) | 1.01(1.00-1.03) | 0.50 |  |  | 0(0) | 1.01(1.00-1.03) | 0.50 |  |
| *2DL3*023* | 10(6) |  | 7(4.5) | 0.74(0.28-2.00) | 0.56 |  |  | 7(4.3) | 0.71(0.26-1.91) | 0.49 |  |
| *2DL3*025* | 1(0.6) |  | 0(0) | 1.01(0.99-1.02) | 1.00 |  |  | 0(0) | 1.01(0.99-1.02) | 1.00 |  |
| *2DL3*026* | 1(0.6) |  | 2(1.3) | 2.17(0.19-24.17) | 0.95 |  |  | 1(0.6) | 1.03(0.06-16.62) | 1.00 |  |
| *2DL3*027* | 1(0.6) |  | 1(0.6) | 1.08(0.07-17.38) | 1.00 |  |  | 1(0.6) | 1.03(0.06-16.62) | 1.00 |  |
| *2DL3*028* | 1(0.6) |  | 0(0) | 1.01(0.99-1.02) | 1.00 |  |  | 1(0.6) | 1.03(0.06-16.62) | 1.00 |  |
| *2DL3*029* | 1(0.6) |  | 0(0) | 1.01(0.99-1.02) | 1.00 |  |  | 0(0) | 1.01(0.99-1.02) | 1.00 |  |
| *2DL3*031* | 1(0.6) |  | 0(0) | 1.01(0.99-1.02) | 1.00 |  |  | 0(0) | 1.01(0.99-1.02) | 1.00 |  |
| *2DL3*032* | 0(0) |  | 1(0.6) | 0.99(0.98-1.01) | 0.48 |  |  | 0(0) | - | - |  |
| *2DL3*037* | 0(0) |  | 1(0.6) | 0.99(0.98-1.01) | 0.48 |  |  | 0(0) | - | - |  |
| *2DS4*00101* | 146(87.4) |  | 136(87.7) | 1.03(0.53-2.00) | 0.93 |  |  | 138(85.2) | 0.83(0.44-1.55) | 0.55 |  |
| *2DS4*00301* | 23(13.8) |  | 23(14.8) | 1.09(0.58-2.04) | 0.78 |  |  | 25(15.4) | 1.14(0.62-2.11) | 0.67 |  |
| *2DS4*00401* | 29(17.4) |  | 28(18.1) | 1.05(0.59-1.86) | 0.87 |  |  | 33(20.4) | 1.22(0.70-2.12) | 0.49 |  |
| *2DS4*010* | 52(31.1) |  | 49(31.6) | 1.02(0.64-1.64) | 0.93 |  |  | 49(30.2) | 0.96(0.60-1.53) | 0.86 |  |
| *2DS4*014* | 0(0) |  | 1(0.6) | 0.99(0.98-1.01) | 0.48 |  |  | 0(0) | - | - |  |
| *3DL1*00101* | 20(12.0) |  | 21(13.5) | 1.15(0.60-2.22) | 0.67 |  |  | 24(14.8) | 1.28(0.68-2.42) | 0.45 |  |
| *3DL1*00501* | 53(31.7) |  | 50(32.3) | 1.02(0.64-1.64) | 0.92 |  |  | 47(29) | 0.88(0.55-1.41) | 0.59 |  |
| *3DL1*00502* | 1(0.6) |  | 0(0) | 1.01(0.99-1.02) | 1.00 |  |  | 0(0) | 1.01(0.99-1.02) | 1.00 |  |
| *3DL1*00701* | 29(17.4) |  | 28(18.1) | 1.05(0.59-1.86) | 0.87 |  |  | 34(21) | 1.26(0.73-2.19) | 0.40 |  |
| *3DL1*008* | 1(0.6) |  | 0(0) | 1.01(0.99-1.02) | 1.00 |  |  | 0(0) | 1.01(0.99-1.02) | 1.00 |  |
| *3DL1*01502* | 139(83.2) |  | 131(84.5) | 1.1(0.61-1.99) | 0.75 |  |  | 130(80.2) | 0.82(0.47-1.43) | 0.48 |  |
| *3DL1*01504* | 0(0) |  | 0(0) | - | - |  |  | 1(0.6) | 0.99(0.98-1.01) | 0.49 |  |
| *3DL1*01505* | 1(0.6) |  | 0(0) | 1.01(0.99-1.02) | 1.00 |  |  | 0(0) | 1.01(0.99-1.02) | 1.00 |  |
| *3DL1*01508* | 0(0) |  | 1(0.6) | 0.99(0.98-1.01) | 0.48 |  |  | 0(0) | - | - |  |
| *3DL1*020* | 14(8.4) |  | 12(7.7) | 0.92(0.41-2.05) | 0.83 |  |  | 10(6.2) | 0.72(0.31-1.67) | 0.44 |  |
| *3DL1*02901* | 5(3.0) |  | 5(3.2) | 1.08(0.31-3.8) | 1.00 |  |  | 4(2.5) | 0.82(0.22-3.11) | 1.00 |  |
| *3DL1*070* | 2(1.2) |  | 0(0) | 1.01(1.00-1.03) | 0.50 |  |  | 0(0) | 1.01(1.00-1.03) | 0.50 |  |
| *3DL1*077* | 0(0) |  | 0(0) | - | - |  |  | 1(0.6) | 0.99(0.98-1.01) | 0.49 |  |
| *3DL1*097* | 0(0) |  | 0(0) | - | - |  |  | 2(1.2) | 0.99(0.97-1.00) | 0.24 |  |
| *3DL1*120* | 0(0) |  | 0(0) | - | - |  |  | 1(0.6) | 0.99(0.98-1.01) | 0.49 |  |
| *3DL1*121* | 0(0) |  | 2(1.3) | 0.99(0.97-1.01) | 0.23 |  |  | 1(0.6) | 0.99(0.98-1.01) | 0.49 |  |
| *2DL4*001* | 147(88.0) |  | 114(86.4) | 0.86(0.44-1.7) | 0.67 |  |  | 132(86.3) | 0.86(0.44-1.65) | 0.64 |  |
| *2DL4*002* | 0(0) |  | 0(0) | - | - |  |  | 1(0.7) | 0.99(0.98-1.01) | 0.48 |  |
| *2DL4*005* | 5(3) |  | 2(1.5) | 0.50(0.10-2.61) | 0.65 |  |  | 5(3.3) | 1.09(0.31-3.86) | 1.00 |  |
| *2DL4*006* | 25(15.0) |  | 27(20.5) | 1.46(0.8-2.66) | 0.21 |  |  | 32(20.9) | 1.5(0.84-2.67) | 0.17 |  |
| *2DL4*008* | 24(14.4) |  | 21(15.9) | 1.13(0.6-2.13) | 0.71 |  |  | 23(15) | 1.05(0.57-1.96) | 0.87 |  |
| *2DL4*011* | 49(29.3) |  | 46(34.8) | 1.29(0.79-2.1) | 0.31 |  |  | 44(28.8) | 0.97(0.6-1.58) | 0.91 |  |
| *2DL4*013* | 0(0) |  | 0(0) | - | - |  |  | 1(0.7) | 0.99(0.98-1.01) | 0.48 |  |
| *2DL4*032* | 1(0.6) |  | 0(0) | 1.01(0.99-1.02) | 1.00 |  |  | 0(0) | 1.01(0.99-1.02) | 1.00 |  |
| *3DL2*001* | 22(13.2) |  | 14(10.6) | 0.78(0.38-1.59) | 0.50 |  |  | 19(12.4) | 0.93(0.48-1.8) | 0.84 |  |
| *3DL2*002* | 139(83.2) |  | 112(84.8) | 1.13(0.60-2.11) | 0.71 |  |  | 121(79.1) | 0.76(0.43-1.34) | 0.34 |  |
| *3DL2*00701* | 2(1.2) |  | 1(0.8) | 0.63(0.06-7.02) | 1.00 |  |  | 2(1.3) | 1.09(0.15-7.85) | 1.00 |  |
| *3DL2*00706* | 0(0) |  | 0(0) | - | - |  |  | 1(0.7) | 0.99(0.98-1.01) | 0.48 |  |
| *3DL2*00707* | 0(0) |  | 0(0) | - | - |  |  | 1(0.7) | 0.99(0.98-1.01) | 0.48 |  |
| *3DL2*008* | 22(13.2) |  | 22(16.7) | 1.32(0.69-2.5) | 0.40 |  |  | 28(18.3) | 1.48(0.80-2.71) | 0.21 |  |
| *3DL2*009* | 14(8.4) |  | 14(10.6) | 1.30(0.60-2.82) | 0.51 |  |  | 8(5.2) | 0.60(0.25-1.48) | 0.27 |  |
| *3DL2*010* | 49(29.3) |  | 35(26.5) | 0.87(0.52-1.45) | 0.59 |  |  | 35(22.9) | 0.71(0.43-1.18) | 0.19 |  |
| *3DL2*015* | 0(0) |  | 3(2.3) | 0.98(0.95-1.00) | 0.17 |  |  | 1(0.7) | 0.99(0.98-1.01) | 0.48 |  |
| *3DL2*016* | 3(1.8) |  | 3(2.3) | 1.27(0.25-6.40) | 1.00 |  |  | 3(2) | 1.09(0.22-5.50) | 1.00 |  |
| *3DL2*021* | 4(2.4) |  | 2(1.5) | 0.63(0.11-3.48) | 0.90 |  |  | 3(2) | 0.82(0.18-3.70) | 1.00 |  |
| *3DL2*036* | 0(0) |  | 0(0) | - | - |  |  | 1(0.7) | 0.99(0.98-1.01) | 0.48 |  |
| *3DL2*039* | 10(6.0) |  | 4(3) | 0.49(0.15-1.60) | 0.23 |  |  | 15(9.8) | 1.71(0.74-3.92) | 0.20 |  |
| *3DL2*063* | 0(0) |  | 0(0) | - | - |  |  | 1(0.7) | 0.99(0.98-1.01) | 0.48 |  |
| *3DL2*083* | 1(0.6) |  | 0(0) | 1.01(0.99-1.02) | 1.00 |  |  | 1(0.7) | 1.09(0.07-17.61) | 1.00 |  |
| *3DL2*084* | 1(0.6) |  | 0(0) | 1.01(0.99-1.02) | 1.00 |  |  | 1(0.7) | 1.09(0.07-17.61) | 1.00 |  |
| *3DL2*091* | 1(0.6) |  | 2(1.5) | 2.55(0.23-28.47) | 0.84 |  |  | 6(3.9) | 6.78(0.81-56.94) | 0.10 |  |
| *3DL2*093* | 1(0.6) |  | 0(0) | 1.01(0.99-1.02) | 1.00 |  |  | 0(0) | 1.01(0.99-1.02) | 1.00 |  |
| *3DL2*099* | 1(0.6) |  | 0(0) | 1.01(0.99-1.02) | 1.00 |  |  | 0(0) | 1.01(0.99-1.02) | 1.00 |  |
| *3DL3*001* | 14(8.4) |  | 5(3.8) | 0.43(0.15-1.23) | 0.11 |  |  | 2(1.3) | 0.14(0.03-0.65) | **0.004** | 0.06 |
| *3DL3*002* | 2(1.2) |  | 3(2.3) | 1.92(0.32-11.65) | 0.79 |  |  | 6(3.9) | 3.37(0.67-16.94) | 0.23 |  |
| *3DL3*006* | 14(8.4) |  | 9(6.8) | 0.80(0.33-1.91) | 0.61 |  |  | 11(7.2) | 0.85(0.37-1.93) | 0.69 |  |
| *3DL3*008* | 51(30.5) |  | 31(23.5) | 0.70(0.42-1.17) | 0.17 |  |  | 42(27.5) | 0.86(0.53-1.4) | 0.54 |  |
| *3DL3*009* | 49(29.3) |  | 59(44.7) | 1.95(1.21-3.14) | **0.01** | 0.08 |  | 72(47.1) | 2.14(1.35-3.39) | **0.001** | **0.016** |
| *3DL3*010* | 125(74.9) |  | 99(75.0) | 1.01(0.60-1.71) | 0.98 |  |  | 102(66.7) | 0.67(0.41-1.09) | 0.11 |  |
| *3DL3*015* | 11(6.6) |  | 11(8.3) | 1.29(0.54-3.07) | 0.57 |  |  | 9(5.9) | 0.89(0.36-2.2) | 0.79 |  |
| *3DL3*02601* | 0(0) |  | 0(0) | - | - |  |  | 1(0.7) | 0.99(0.98-1.01) | 0.48 |  |
| *3DL3*02602* | 3(1.8) |  | 1(0.8) | 0.42(0.04-4.06) | 0.79 |  |  | 3(2) | 1.09(0.22-5.5) | 1.00 |  |
| *3DL3*04801* | 3(1.8) |  | 0(0) | 1.02(1.00-1.04) | 0.34 |  |  | 0(0) | 1.02(1.00-1.04) | 0.28 |  |
| *3DL3*04802* | 8(4.8) |  | 4(3.0) | 0.62(0.18-2.11) | 0.44 |  |  | 5(3.3) | 0.67(0.21-2.10) | 0.49 |  |
| *3DL3*062* | 3(1.8) |  | 0(0) | 1.02(1.00-1.04) | 0.34 |  |  | 3(2) | 1.09(0.22-5.50) | 1.00 |  |
| *3DL3*063* | 2(1.2) |  | 2(1.5) | 1.27(0.18-9.13) | 1.00 |  |  | 0(0) | 1.01(1.00-1.03) | 0.50 |  |
| *3DL3*064* | 2(1.2) |  | 4(3.0) | 2.58(0.46-14.3) | 0.48 |  |  | 2(1.3) | 1.09(0.15-7.85) | 1.00 |  |
| *3DL3*065* | 2(1.2) |  | 1(0.8) | 0.63(0.06-7.02) | 1.00 |  |  | 2(1.3) | 1.09(0.15-7.85) | 1.00 |  |

**Table S4**  Comparison analysis of the observed frequencies for *KIR* alleles between healthy controls and ALL/AML patient groups having the *KIR Bx* genotype.

| Allele | Control（n=139） |  | ALL (n=163) | | |  | AML (n=174) | | |
| --- | --- | --- | --- | --- | --- | --- | --- | --- | --- |
|  | n (%) |  | n (%) | OR (95% CI) | *P* |  | n (%) | OR (95% CI) | *P* |
| *2DL1*001* | 1(0.7) |  | 0(0) | 1.01(0.99-1.02) | 0.46 |  | 0(0) | 1.01(0.99-1.02) | 0.44 |
| *2DL1*00201* | 28(20.1) |  | 35(21.5) | 1.08(0.62-1.89) | 0.78 |  | 49(28.2) | 1.55(0.91-2.64) | 0.10 |
| *2DL1*00302* | 121(87.1) |  | 135(82.8) | 0.72(0.38-1.36) | 0.31 |  | 147(84.5) | 0.81(0.43-1.54) | 0.52 |
| *2DL1*00304* | 1(0.7) |  | 0(0) | 1.01(0.99-1.02) | 0.46 |  | 0(0) | 1.01(0.99-1.02) | 0.44 |
| *2DL1*00305* | 1(0.7) |  | 0(0) | 1.01(0.99-1.02) | 0.46 |  | 1(0.6) | 0.80(0.05-12.87) | 1.00 |
| *2DL1*004* | 19(13.7) |  | 33(20.2) | 1.60(0.87-2.97) | 0.13 |  | 20(11.5) | 0.82(0.42-1.61) | 0.56 |
| *2DL1*010* | 0(0) |  | 1(0.6) | 0.99(0.98-1.01) | 1.00 |  | 0(0) | - | - |
| *2DL1*020* | 0(0) |  | 1(0.6) | 0.99(0.98-1.01) | 1.00 |  | 0(0) | - | - |
| *2DL1*031* | 1(0.7) |  | 0(0) | 1.01(0.99-1.02) | 0.46 |  | 0(0) | 1.01(0.99-1.02) | 0.44 |
| *2DL1*034* | 0(0) |  | 2(1.2) | 0.99(0.97-1.00) | 0.50 |  | 1(0.6) | 0.99(0.98-1.01) | 1.00 |
| *2DL1*069* | 0(0) |  | 1(0.6) | 0.99(0.98-1.01) | 1.00 |  | 4(2.3) | 0.98(0.95-1.00) | 0.20 |
| *2DL1*070* | 0(0) |  | 2(1.2) | 0.99(0.97-1.00) | 0.50 |  | 1(0.6) | 0.99(0.98-1.01) | 1.00 |
| *2DL1*071* | 0(0) |  | 1(0.6) | 0.99(0.98-1.01) | 1.00 |  | 0(0) | - | - |
| *2DL3*00101* | 123(88.5) |  | 135(82.8) | 0.63(0.32-1.21) | 0.16 |  | 149(85.6) | 0.78(0.40-1.52) | 0.46 |
| *2DL3*00103* | 0(0) |  | 0(0) | - | - |  | 1(0.6) | 0.99(0.98-1.01) | 1.00 |
| *2DL3*00105* | 0(0) |  | 0(0) | - | - |  | 1(0.6) | 0.99(0.98-1.01) | 1.00 |
| *2DL3*00109* | 3(2.2) |  | 1(0.6) | 0.28(0.03-2.72) | 0.51 |  | 0(0) | 1.02(1.00-1.05) | 0.17 |
| *2DL3*00110* | 1(0.7) |  | 0(0) | 1.01(0.99-1.02) | 0.46 |  | 0(0) | 1.01(0.99-1.02) | 0.44 |
| *2DL3*00112* | 0(0) |  | 1(0.6) | 0.99(0.98-1.01) | 1.00 |  | 0(0) | - | - |
| *2DL3*00201* | 27(19.4) |  | 34(20.9) | 1.09(0.62-1.92) | 0.76 |  | 46(26.4) | 1.49(0.87-2.55) | 0.14 |
| *2DL3*015* | 1(0.7) |  | 3(1.8) | 2.59(0.27-25.16) | 0.73 |  | 4(2.3) | 3.25(0.36-29.39) | 0.51 |
| *2DL3*019* | 0(0) |  | 2(1.2) | 0.99(0.97-1.00) | 0.50 |  | 0(0) | - | - |
| *2DL3*022* | 0(0) |  | 0(0) | - | - |  | 2(1.1) | 0.99(0.97-1.00) | 0.50 |
| *2DL3*023* | 3(2.2) |  | 3(1.8) | 0.85(0.17-4.28) | 1.00 |  | 2(1.1) | 0.53(0.09-3.20) | 0.80 |
| *2DL3*026* | 0(0) |  | 2(1.2) | 0.99(0.97-1.00) | 0.50 |  | 2(1.1) | 0.99(0.97-1.00) | 0.50 |
| *2DL3*027* | 0(0) |  | 0(0) | - | - |  | 2(1.1) | 0.99(0.97-1.00) | 0.50 |
| *2DL3*030* | 0(0) |  | 1(0.6) | 0.99(0.98-1.01) | 1.00 |  | 0(0) | - | - |
| *2DL3*032* | 0(0) |  | 1(0.6) | 0.99(0.98-1.01) | 1.00 |  | 1(0.6) | 0.99(0.98-1.01) | 1.00 |
| *2DS4*00101* | 95(68.3) |  | 107(65.6) | 0.88(0.55-1.43) | 0.62 |  | 106(60.9) | 0.72(0.45-1.15) | 0.17 |
| *2DS4*00105* | 1(0.7) |  | 0(0) | 1.01(0.99-1.02) | 0.46 |  | 0(0) | 1.01(0.99-1.02) | 0.44 |
| *2DS4*00301* | 9(6.5) |  | 18(11.0) | 1.79(0.78-4.13) | 0.17 |  | 12(6.9) | 1.07(0.44-2.62) | 0.88 |
| *2DS4*00401* | 20(14.4) |  | 18(11.0) | 0.74(0.37-1.46) | 0.38 |  | 19(10.9) | 0.73(0.37-1.43) | 0.36 |
| *2DS4*010* | 19(13.7) |  | 36(22.1) | 1.79(0.97-3.29) | 0.06 |  | 38(21.8) | 1.76(0.97-3.22) | 0.06 |
| *2DS4*014* | 0(0) |  | 1(0.6) | 0.99(0.98-1.01) | 1.00 |  | 0(0) | - | - |
| *2DS4*017* | 1(0.7) |  | 0(0) | 1.01(0.99-1.02) | 0.46 |  | 0(0) | 1.01(0.99-1.02) | 0.44 |
| *2DS4*018* | 2(1.4) |  | 0(0) | 1.01(0.99-1.04) | 0.21 |  | 0(0) | 1.01(0.99-1.04) | 0.20 |
| *3DL1*00101* | 10(7.2) |  | 17(10.4) | 1.50(0.66-3.40) | 0.33 |  | 11(6.3) | 0.87(0.36-2.11) | 0.76 |
| *3DL1*00501* | 20(14.4) |  | 36(22.1) | 1.69(0.92-3.08) | 0.09 |  | 38(21.8) | 1.66(0.92-3.01) | 0.09 |
| *3DL1*00502* | 0(0) |  | 0(0) | - | - |  | 2(1.1) | 0.99(0.97-1.00) | 0.50 |
| *3DL1*00701* | 20(14.4) |  | 18(11.0) | 0.74(0.37-1.46) | 0.38 |  | 19(10.9) | 0.73(0.37-1.43) | 0.36 |
| *3DL1*008* | 0(0) |  | 1(0.6) | 0.99(0.98-1.01) | 1.00 |  | 0(0) | - | - |
| *3DL1*01502* | 89(64.0) |  | 101(62) | 0.92(0.57-1.46) | 0.71 |  | 96(55.2) | 0.69(0.44-1.09) | 0.11 |
| *3DL1*020* | 10(7.2) |  | 5(3.1) | 0.41(0.14-1.22) | 0.10 |  | 9(5.2) | 0.70(0.28-1.78) | 0.46 |
| *3DL1*02901* | 5(3.6) |  | 2(1.2) | 0.33(0.06-1.74) | 0.33 |  | 2(1.1) | 0.31(0.06-1.63) | 0.28 |
| *3DL1*070* | 0(0) |  | 1(0.6) | 0.99(0.98-1.01) | 1.00 |  | 0(0) | - | - |
| *3DL1*077* | 0(0) |  | 0(0) | - | - |  | 4(2.3) | 0.98(0.95-1.00) | 0.20 |
| *3DL1*079* | 1(0.7) |  | 0(0) | 1.01(0.99-1.02) | 0.46 |  | 0(0) | 1.01(0.99-1.02) | 0.44 |
| *3DL1*097* | 0(0) |  | 1(0.6) | 0.99(0.98-1.01) | 1.00 |  | 2(1.1) | 0.99(0.97-1.00) | 0.50 |
| *3DL1*121* | 0(0) |  | 0(0) | - | - |  | 1(0.6) | 0.99(0.98-1.01) | 1.00 |
| *2DL4*001* | 96(69.1) |  | 91(66.4) | 0.89(0.53-1.47) | 0.64 |  | 103(62.8) | 0.76(0.47-1.22) | 0.25 |
| *2DL4*005* | 103(74.1) |  | 90(65.7) | 0.67(0.4-1.12) | 0.13 |  | 126(76.8) | 1.16(0.69-1.96) | 0.58 |
| *2DL4*006* | 19(13.7) |  | 13(9.5) | 0.66(0.31-1.40) | 0.28 |  | 17(10.4) | 0.73(0.36-1.47) | 0.38 |
| *2DL4*008* | 11(7.9) |  | 15(10.9) | 1.43(0.63-3.24) | 0.39 |  | 11(6.7) | 0.84(0.35-1.99) | 0.69 |
| *2DL4*011* | 19(13.7) |  | 31(22.6) | 1.85(0.99-3.46) | 0.05 |  | 36(22) | 1.78(0.97-3.27) | 0.06 |
| *2DL4*013* | 0(0) |  | 0(0) | - | - |  | 1(0.6) | 0.99(0.98-1.01) | 1.00 |
| *2DL4*033* | 1(0.7) |  | 1(0.7) | 1.01(0.06-16.39) | 1.00 |  | 0(0) | 1.01(0.99-1.02) | 0.46 |
| *2DL4*034* | 1(0.7) |  | 0(0) | 0.99(0.98-1.01) | 1.00 |  | 1(0.6) | 0.85(0.05-13.66) | 1.00 |
| *3DL2*001* | 13(9.4) |  | 16(11.7) | 1.28(0.59-2.78) | 0.53 |  | 11(6.7) | 0.70(0.30-1.61) | 0.40 |
| *3DL2*002* | 99(71.2) |  | 104(75.9) | 1.27(0.74-2.18) | 0.38 |  | 120(73.2) | 1.1(0.67-1.82) | 0.71 |
| *3DL2*00701* | 60(43.2) |  | 52(38.0) | 0.81(0.50-1.30) | 0.38 |  | 78(47.6) | 1.19(0.76-1.88) | 0.44 |
| *3DL2*00702* | 0(0) |  | 0(0) | - | - |  | 1(0.6) | 0.99(0.98-1.01) | 1.00 |
| *3DL2*00706* | 2(1.4) |  | 4(2.9) | 2.06(0.37-11.44) | 0.67 |  | 0(0) | 1.01(0.99-1.04) | 0.21 |
| *3DL2*00707* | 2(1.4) |  | 2(1.5) | 1.01(0.14-7.31) | 1.00 |  | 1(0.6) | 0.42(0.04-4.68) | 0.89 |
| *3DL2*008* | 17(12.2) |  | 8(5.8) | 0.45(0.19-1.07) | 0.06 |  | 12(7.3) | 0.57(0.26-1.23) | 0.15 |
| *3DL2*009* | 8(5.8) |  | 2(1.5) | 0.24(0.05-1.16) | 0.11 |  | 6(3.7) | 0.62(0.21-1.84) | 0.39 |
| *3DL2*010* | 19(13.7) |  | 25(18.2) | 1.41(0.74-2.70) | 0.30 |  | 27(16.5) | 1.24(0.66-2.35) | 0.50 |
| *3DL2*015* | 6(4.3) |  | 1(0.7) | 0.16(0.02-1.37) | 0.13 |  | 4(2.4) | 0.55(0.15-2.00) | 0.56 |
| *3DL2*016* | 0(0) |  | 0(0) | - | - |  | 3(1.8) | 0.98(0.96-1.00) | 0.31 |
| *3DL2*021* | 2(1.4) |  | 2(1.5) | 1.01(0.14-7.31) | 1.00 |  | 3(1.8) | 1.28(0.21-7.75) | 1.00 |
| *3DL2*027* | 2(1.4) |  | 0(0) | 1.01(0.99-1.04) | 0.50 |  | 1(0.6) | 0.42(0.04-4.68) | 0.89 |
| *3DL2*036* | 0(0) |  | 0(0) | - | - |  | 1(0.6) | 0.99(0.98-1.01) | 1.00 |
| *3DL2*039* | 9(6.5) |  | 6(4.4) | 0.66(0.23-1.91) | 0.44 |  | 4(2.4) | 0.36(0.11-1.20) | 0.08 |
| *3DL2*047* | 0(0) |  | 0(0) | - | - |  | 1(0.6) | 0.99(0.98-1.01) | 1.00 |
| *3DL2*083* | 0(0) |  | 2(1.5) | 0.99(0.97-1.01) | 0.25 |  | 0(0) | - | - |
| *3DL2*084* | 0(0) |  | 1(0.7) | 0.99(0.98-1.01) | 0.50 |  | 0(0) | - | - |
| *3DL2*091* | 2(1.4) |  | 4(2.9) | 2.06(0.37-11.44) | 0.67 |  | 2(1.2) | 0.85(0.12-6.08) | 1.00 |
| *3DL2*093* | 5(3.6) |  | 6(4.4) | 1.23(0.37-4.12) | 0.74 |  | 15(9.1) | 2.70(0.95-7.62) | 0.05 |
| *3DL3*001* | 10(7.2) |  | 7(5.1) | 0.69(0.26-1.88) | 0.47 |  | 9(5.5) | 0.75(0.30-1.90) | 0.54 |
| *3DL3*002* | 1(0.7) |  | 2(1.5) | 2.04(0.18-22.81) | 0.99 |  | 4(2.4) | 3.45(0.38-31.23) | 0.47 |
| *3DL3*003* | 3(2.2) |  | 0(0) | 1.02(1.00-1.05) | 0.25 |  | 3(1.8) | 0.84(0.17-4.25) | 1.00 |
| *3DL3*004* | 5(3.6) |  | 7(5.1) | 1.44(0.45-4.66) | 0.54 |  | 8(4.9) | 1.37(0.44-4.3) | 0.58 |
| *3DL3*006* | 14(10.1) |  | 12(8.8) | 0.86(0.38-1.93) | 0.71 |  | 23(14) | 1.46(0.72-2.95) | 0.30 |
| *3DL3*008* | 47(33.8) |  | 38(27.7) | 0.75(0.45-1.26) | 0.27 |  | 39(23.8) | 0.61(0.37-1.01) | 0.05 |
| *3DL3*009* | 36(25.9) |  | 42(30.7) | 1.26(0.75-2.14) | 0.38 |  | 51(31.1) | 1.29(0.78-2.14) | 0.32 |
| *3DL3*010* | 87(62.6) |  | 83(60.6) | 0.92(0.57-1.49) | 0.73 |  | 103(62.8) | 1.01(0.63-1.61) | 0.97 |
| *3DL3*01003* | 1(0.7) |  | 0(0) | 1.01(0.99-1.02) | 1.00 |  | 0(0) | 1.01(0.99-1.02) | 0.46 |
| *3DL3*013* | 1(0.7) |  | 0(0) | 1.01(0.99-1.02) | 1.00 |  | 0(0) | 1.01(0.99-1.02) | 0.46 |
| *3DL3*015* | 9(6.5) |  | 10(7.3) | 1.14(0.45-2.89) | 0.79 |  | 11(6.7) | 1.04(0.42-2.58) | 0.94 |
| *3DL3*02601* | 0(0) |  | 2(1.5) | 0.99(0.97-1.01) | 0.25 |  | 1(0.6) | 0.99(0.98-1.01) | 1.00 |
| *3DL3*02602* | 3(2.2) |  | 6(4.4) | 2.08(0.51-8.48) | 0.48 |  | 4(2.4) | 1.13(0.25-5.15) | 1.00 |
| *3DL3*028* | 10(7.2) |  | 10(7.3) | 1.02(0.41-2.52) | 0.97 |  | 9(5.5) | 0.75(0.30-1.90) | 0.54 |
| *3DL3*04801* | 3(2.2) |  | 1(0.7) | 0.33(0.03-3.24) | 0.62 |  | 6(3.7) | 1.72(0.42-7.01) | 0.67 |
| *3DL3*04802* | 4(2.9) |  | 9(6.6) | 2.37(0.71-7.9) | 0.15 |  | 13(7.9) | 2.91(0.93-9.13) | 0.06 |
| *3DL3*062* | 0(0) |  | 1(0.7) | 0.99(0.98-1.01) | 0.50 |  | 2(1.2) | 0.99(0.97-1.00) | 0.50 |
| *3DL3*063* | 0(0) |  | 4(2.9) | 0.97(0.94-1.00) | 0.13 |  | 1(0.6) | 0.99(0.98-1.01) | 1.00 |
| *3DL3*064* | 1(0.7) |  | 0(0) | 1.01(0.99-1.02) | 1.00 |  | 1(0.6) | 0.85(0.05-13.66) | 1.00 |
| *3DL3*065* | 0(0) |  | 0(0) | - | - |  | 1(0.6) | 0.99(0.98-1.01) | 1.00 |

**Table S5**  Comparison analysis of the allele frequencies for *KIR* allotypes between healthy controls and ALL/AML patient groups having the *KIR AA* genotype.

| Allele | Control (N=167) |  | ALL (n=155) | | | |  | AML(n=162) | | | |
| --- | --- | --- | --- | --- | --- | --- | --- | --- | --- | --- | --- |
|  | n (%) |  | n (%) | OR (95% CI) | *P* | *P_c_* |  | n (%) | OR (95% CI) | *P* | *P_c_* |
| 2DL1_R245 | 334(100) |  | 310(100) | - | - |  |  | 323(99.6) | 0.99(0.99-1) | 0.49 |  |
| 2DL1_C245 | 0(0) |  | 0(0) | - | - |  |  | 1(0.3) | 0.99(0.99-1) | 0.49 |  |
| 2DL1_P114 | 50(14.9) |  | 38(12.2) | 0.79(0.5-1.24) | 0.32 |  |  | 32(9.8) | 0.62(0.38-0.99) | 0.05 |  |
| 2DL1_L114 | 284(85) |  | 272(87.7) | 1.26(0.8-1.98) | 0.32 |  |  | 292(90.1) | 1.6(1-2.57) | 0.05 |  |
| 2DS4 deleted | 112(33.5) |  | 108(34.8) | 1.05(0.76-1.46) | 0.73 |  |  | 121(37.3) | 1.18(0.85-1.62) | 0.31 |  |
| 2DS4 normal | 222(66.4) |  | 202(65.1) | 0.94(0.68-1.3) | 0.73 |  |  | 203(62.6) | 0.84(0.61-1.16) | 0.31 |  |
| 2DL4-10A | 254(76) |  | 190(71.9) | 0.8(0.55-1.16) | 0.26 |  |  | 231(75.4) | 0.97(0.67-1.39) | 0.87 |  |
| 2DL4-9A | 80(23.9) |  | 74(28) | 1.23(0.85-1.78) | 0.26 |  |  | 75(24.5) | 1.03(0.71-1.48) | 0.87 |  |
| KIR3DL1*h | 237(70.9) |  | 218(70.3) | 0.96(0.69-1.36) | 0.86 |  |  | 225(69.4) | 0.93(0.66-1.29) | 0.67 |  |
| KIR3DL1*l | 90(26.9) |  | 85(27.4) | 1.02(0.72-1.44) | 0.89 |  |  | 90(27.7) | 1.04(0.74-1.46) | 0.81 |  |
| 3DL3_N300 | 51(15.2) |  | 27(10.2) | 0.63(0.38-1.03) | 0.07 |  |  | 29(9.4) | 0.58(0.35-0.94) | **0.03** | 0.05 |
| 3DL3_H300 | 283(84.7) |  | 237(89.7) | 1.58(0.96-2.6) | 0.07 |  |  | 277(90.5) | 1.72(1.05-2.79) | **0.03** | 0.05 |
| 3DL3_Y300 | 0(0) |  | 0(0) | - | - |  |  | 0(0) | - | - |  |

Table S6. Comparison analysis of the observed frequencies for *HLA* ligands between healthy controls and ALL/AML patient groups having the *KIR AA* genotype.

| *HLA* Ligand | Control (n=167) |  | ALL (n=155) | | | |  | AML (n=162) | | | |
| --- | --- | --- | --- | --- | --- | --- | --- | --- | --- | --- | --- |
|  | n (%) |  | n (%) | OR (95% CI) | P | Pc |  | n (%) | OR (95% CI) | P | Pc |
| *HLA-A3/A11* | 101(60.5) |  | 89(57.4) | 0.88(0.56-1.37) | 0.58 |  |  | 90(55.6) | 0.82(0.53-1.27) | 0.37 |  |
| *HLA-Bw4* | 123(73.7) |  | 113(72.9) | 0.96(0.59-1.58) | 0.88 |  |  | 115(71.0) | 0.88(0.54-1.42) | 0.59 |  |
| *HLA-A Bw4* | 51(30.5) |  | 43(27.7) | 0.87(0.54-1.41) | 0.58 |  |  | 60(37.0) | 1.34(0.85-2.12) | 0.21 |  |
| *HLA-B Bw4 80I* | 59(35.3) |  | 47(30.3) | 0.80(0.50-1.27) | 0.34 |  |  | 42(25.9) | 0.64(0.40-1.03) | 0.06 |  |
| *HLA-B Bw4 80T* | 55(32.9) |  | 50(32.3) | 0.97(0.61-1.55) | 0.90 |  |  | 51(31.5) | 0.94(0.59-1.49) | 0.78 |  |
| *HLA-C1* | 164(98.2) |  | 152(98.1) | 0.93(0.18-4.66) | 1.00 |  |  | 160(98.8) | 1.46(0.24-8.87) | 1.00 |  |
| *HLA-C2* | 52(31.1) |  | 39(25.2) | 0.74(0.46-1.21) | 0.23 |  |  | 29(17.9) | 0.48(0.29-0.81) | **0.005** | **0.01** |

**Table S7**  Comparison analysis of the observed frequencies for *KIR* allele+*HLA* interactions between healthy controls and ALL/AML patient groups having the *KIR AA* genotype.

| *KIR+HLA* Ligand | Control (n=167) |  | ALL (n=155) | | | |  | AML (n=162) | | | |
| --- | --- | --- | --- | --- | --- | --- | --- | --- | --- | --- | --- |
|  | n (%) |  | n (%) | OR (95% CI) | *P* | *P*_c_ |  | n (%) | OR (95% CI) | *P* | *P*_c_ |
| *2DL1*00201+C2* | 15(9.0) |  | 4(2.6) | 0.27(0.09-0.83) | **0.01** | 1.06 |  | 7(4.3) | 0.46(0.18-1.15) | 0.09 |  |
| *2DL1*00302+C2* | 51(30.5) |  | 39(25.2) | 0.76(0.47-1.25) | 0.28 |  |  | 29(17.9) | 0.50(0.30-0.83) | **0.008** | 0.62 |
| *2DL1*00309+C2* | 0(0) |  | 0(0) | - | - |  |  | 1(0.6) | 0.99(0.98-1.01) | 0.49 |  |
| *2DL1*034+C2* | 0(0) |  | 1(0.6) | 0.99(0.98-1.01) | 0.48 |  |  | 0(0) | - | - |  |
| *2DL1*069+C2* | 0(0) |  | 2(1.3) | 0.99(0.97-1.01) | 0.23 |  |  | 1(0.6) | 0.99(0.98-1.01) | 0.49 |  |
| *2DL1*074+C2* | 0(0) |  | 1(0.6) | 0.99(0.98-1.01) | 0.48 |  |  | 0(0) | - | - |  |
| *2DL3*00101+C1* | 157(94.0) |  | 146(94.2) | 1.03(0.41-2.61) | 0.94 |  |  | 157(96.9) | 2.00(0.67-5.98) | 0.21 |  |
| *2DL3*00103+C1* | 0(0) |  | 1(0.6) | 0.99(0.98-1.01) | 0.48 |  |  | 1(0.6) | 0.99(0.98-1.01) | 0.49 |  |
| *2DL3*00108+C1* | 0(0) |  | 0(0) | - | - |  |  | 2(1.2) | 0.99(0.97-1.00) | 0.24 |  |
| *2DL3*00109+C1* | 1(0.6) |  | 1(0.6) | 1.08(0.07-17.38) | 1.00 |  |  | 0(0) | 1.01(0.99-1.02) | 1.00 |  |
| *2DL3*00110+C1* | 0(0) |  | 0(0) | - | - |  |  | 1(0.6) | 0.99(0.98-1.01) | 0.49 |  |
| *2DL3*00111+C1* | 0(0) |  | 1(0.6) | 0.99(0.98-1.01) | 0.48 |  |  | 0(0) | - | - |  |
| *2DL3*00112+C1* | 0(0) |  | 0(0) | - | - |  |  | 1(0.6) | 0.99(0.98-1.01) | 0.49 |  |
| *2DL3*00201+C1* | 47(28.1) |  | 33(21.3) | 0.69(0.41-1.15) | 0.16 |  |  | 29(17.9) | 0.56(0.33-0.94) | **0.03** | 2.26 |
| *2DL3*015+C1* | 1(0.6) |  | 2(1.3) | 2.17(0.19-24.17) | 0.95 |  |  | 2(1.2) | 2.08(0.19-23.11) | 0.98 |  |
| *2DL3*019+C1* | 1(0.6) |  | 1(0.6) | 1.08(0.07-17.38) | 1.00 |  |  | 2(1.2) | 2.08(0.19-23.11) | 0.98 |  |
| *2DL3*021+C1* | 1(0.6) |  | 0(0) | 1.01(0.99-1.02) | 1.00 |  |  | 0(0) | 1.01(0.99-1.02) | 1.00 |  |
| *2DL3*022+C1* | 2(1.2) |  | 0(0) | 1.01(1.00-1.03) | 0.50 |  |  | 0(0) | 1.01(1.00-1.03) | 0.50 |  |
| *2DL3*023+C1* | 10(6.0) |  | 7(4.5) | 0.74(0.28-2.00) | 0.56 |  |  | 7(4.3) | 0.71(0.26-1.91) | 0.49 |  |
| *2DL3*025+C1* | 1(0.6) |  | 0(0) | 1.01(0.99-1.02) | 1.00 |  |  | 0(0) | 1.01(0.99-1.02) | 1.00 |  |
| *2DL3*026+C1* | 1(0.6) |  | 2(1.3) | 2.17(0.19-24.17) | 0.95 |  |  | 1(0.6) | 1.03(0.06-16.62) | 1.00 |  |
| *2DL3*027+C1* | 1(0.6) |  | 1(0.6) | 1.08(0.07-17.38) | 1.00 |  |  | 1(0.6) | 1.03(0.06-16.62) | 1.00 |  |
| *2DL3*028+C1* | 1(0.6) |  | 0(0) | 1.01(0.99-1.02) | 1.00 |  |  | 1(0.6) | 1.03(0.06-16.62) | 1.00 |  |
| *2DL3*029+C1* | 1(0.6) |  | 0(0) | 1.01(0.99-1.02) | 1.00 |  |  | 0(0) | 1.01(0.99-1.02) | 1.00 |  |
| *2DL3*031+C1* | 1(0.6) |  | 0(0) | 1.01(0.99-1.02) | 1.00 |  |  | 0(0) | 1.01(0.99-1.02) | 1.00 |  |
| *2DL3*032+C1* | 0(0) |  | 1(0.6) | 0.99(0.98-1.01) | 0.48 |  |  | 0(0) | - | - |  |
| *3DL1*00101+Bw4* | 14(8.4) |  | 13(8.4) | 1.00(0.45-2.20) | 1.00 |  |  | 19(11.7) | 1.45(0.70-3.00) | 0.31 |  |
| *3DL1*00501+Bw4* | 41(24.6) |  | 37(23.9) | 0.96(0.58-1.61) | 0.89 |  |  | 34(21.0) | 0.82(0.49-1.37) | 0.44 |  |
| *3DL1*00502+Bw4* | 1(0.6) |  | 0(0) | 1.01(0.99-1.02) | 1.00 |  |  | 0(0) | 1.01(0.99-1.02) | 1.00 |  |
| *3DL1*00701+Bw4* | 23(13.8) |  | 19(12.3) | 0.87(0.46-1.68) | 0.69 |  |  | 22(13.6) | 0.98(0.52-1.85) | 0.96 |  |
| *3DL1*008+Bw4* | 1(0.6) |  | 0(0) | 1.01(0.99-1.02) | 1.00 |  |  | 0(0) | 1.01(0.99-1.02) | 1.00 |  |
| *3DL1*01502+Bw4* | 102(61.1) |  | 93(60.0) | 0.96(0.61-1.49) | 0.84 |  |  | 92(56.8) | 0.84(0.54-1.30) | 0.43 |  |
| *3DL1*01504+Bw4* | 0(0) |  | 0(0) | - | - |  |  | 1(0.6) | 0.99(0.98-1.01) | 0.49 |  |
| *3DL1*01505+Bw4* | 1(0.6) |  | 0(0) | 1.01(0.99-1.02) | 1.00 |  |  | 0(0) | 1.01(0.99-1.02) | 1.00 |  |
| *3DL1*020+Bw4* | 11(6.6) |  | 11(7.1) | 1.08(0.46-2.58) | 0.86 |  |  | 6(3.7) | 0.55(0.20-1.51) | 0.24 |  |
| *3DL1*02901+Bw4* | 3(1.8) |  | 4(2.6) | 1.45(0.32-6.58) | 0.92 |  |  | 2(1.2) | 0.68(0.11-4.14) | 1.00 |  |
| *3DL1*070+Bw4* | 1(0.6) |  | 0(0) | 1.01(0.99-1.02) | 1.00 |  |  | 0(0) | 1.01(0.99-1.02) | 1.00 |  |
| *3DL1*077+Bw4* | 0(0) |  | 0(0) | - | - |  |  | 1(0.6) | 0.99(0.98-1.01) | 0.49 |  |
| *3DL1*097+Bw4* | 0(0) |  | 0(0) | - | - |  |  | 2(1.2) | 0.99(0.97-1.00) | 0.24 |  |
| *3DL1*121+Bw4* | 0(0) |  | 1(0.6) | 0.99(0.98-1.01) | 0.48 |  |  | 1(0.6) | 0.99(0.98-1.01) | 0.49 |  |
| *3DL1*00101+Bw4 80I* | 8(4.8) |  | 3(1.9) | 0.39(0.10-1.51) | 0.16 |  |  | 7(4.3) | 0.9(0.32-2.53) | 0.84 |  |
| *3DL1*00501+Bw4 80I* | 24(14.4) |  | 15(9.7) | 0.64(0.32-1.27) | 0.20 |  |  | 9(5.6) | 0.35(0.16-0.78) | **0.008** | 0.64 |
| *3DL1*00701+Bw4 80I* | 11(6.6) |  | 6(3.9) | 0.57(0.21-1.58) | 0.28 |  |  | 7(4.3) | 0.64(0.24-1.70) | 0.37 |  |
| *3DL1*008+Bw4 80I* | 1(0.6) |  | 0(0) | 1.01(0.99-1.02) | 1.00 |  |  | 0(0) | 1.01(0.99-1.02) | 1.00 |  |
| *3DL1*01502+Bw4 80I* | 49(29.3) |  | 41(26.5) | 0.87(0.53-1.41) | 0.56 |  |  | 35(21.6) | 0.66(0.40-1.10) | 0.11 |  |
| *3DL1*01505+Bw4 80I* | 1(0.6) |  | 0(0) | 1.01(0.99-1.02) | 1.00 |  |  | 0(0) | 1.01(0.99-1.02) | 1.00 |  |
| *3DL1*020+Bw4 80I* | 0(0) |  | 5(3.2) | 0.97(0.94-1.00) | 0.06 |  |  | 1(0.6) | 0.99(0.98-1.01) | 0.49 |  |
| *3DL1*02901+Bw4 80I* | 2(1.2) |  | 1(0.6) | 0.54(0.05-5.97) | 1.00 |  |  | 1(0.6) | 0.51(0.05-5.71) | 1.00 |  |
| *3DL1*070+Bw4 80I* | 1(0.6) |  | 0(0) | 1.01(0.99-1.02) | 1.00 |  |  | 0(0) | 1.01(0.99-1.02) | 1.00 |  |
| *3DL1*077+Bw4 80I* | 0(0) |  | 0(0) | - | - |  |  | 1(0.6) | 0.99(0.98-1.01) | 0.49 |  |
| *3DL1*097+Bw4 80I* | 0(0) |  | 0(0) | - | - |  |  | 1(0.6) | 0.99(0.98-1.01) | 0.49 |  |
| *3DL1*121+Bw4 80I* | 0(0) |  | 1(0.6) | 0.99(0.98-1.01) | 0.48 |  |  | 1(0.6) | 0.99(0.98-1.01) | 0.49 |  |
| *3DL1*00101+Bw4 80T* | 4(2.4) |  | 7(4.5) | 1.93(0.55-6.72) | 0.30 |  |  | 11(6.8) | 2.97(0.93-9.52) | 0.06 |  |
| *3DL1*00501+Bw4 80T* | 17(10.2) |  | 11(7.1) | 0.67(0.31-1.49) | 0.33 |  |  | 15(9.3) | 0.90(0.43-1.87) | 0.78 |  |
| *3DL1*00502+Bw4 80T* | 1(0.6) |  | 0(0) | 1.01(0.99-1.02) | 1.00 |  |  | 0(0) | 1.01(0.99-1.02) | 1.00 |  |
| *3DL1*00701+Bw4 80T* | 12(7.2) |  | 8(5.2) | 0.70(0.28-1.77) | 0.45 |  |  | 10(6.2) | 0.85(0.36-2.03) | 0.71 |  |
| *3DL1*01502+Bw4 80T* | 45(26.9) |  | 42(27.1) | 1.01(0.62-1.65) | 0.98 |  |  | 42(25.9) | 0.95(0.58-1.55) | 0.83 |  |
| *3DL1*020+Bw4 80T* | 6(3.6) |  | 4(2.6) | 0.71(0.2-2.57) | 0.84 |  |  | 3(1.9) | 0.51(0.12-2.06) | 0.53 |  |
| *3DL1*02901+Bw4 80T* | 1(0.6) |  | 2(1.3) | 2.17(0.19-24.17) | 0.95 |  |  | 1(0.6) | 1.03(0.06-16.62) | 1.00 |  |
| *3DL1*121+Bw4 80T* | 0(0) |  | 1(0.6) | 0.99(0.98-1.01) | 0.48 |  |  | 0(0) | - | - |  |
| *3DL1*00101+A Bw4* | 8(4.8) |  | 4(2.6) | 0.53(0.16-1.78) | 0.30 |  |  | 8(4.9) | 1.03(0.38-2.82) | 0.95 |  |
| *3DL1*00501+A Bw4* | 15(9.0) |  | 18(11.6) | 1.33(0.65-2.74) | 0.44 |  |  | 21(13.0) | 1.51(0.75-3.04) | 0.25 |  |
| *3DL1*00502+A Bw4* | 1(0.6) |  | 0(0) | 1.01(0.99-1.02) | 1.00 |  |  | 0(0) | 1.01(0.99-1.02) | 1.00 |  |
| *3DL1*00701+A Bw4* | 10(6.0) |  | 9(5.8) | 0.97(0.38-2.45) | 0.94 |  |  | 11(6.8) | 1.14(0.47-2.77) | 0.77 |  |
| *3DL1*008+A Bw4* | 1(0.6) |  | 0(0) | 1.01(0.99-1.02) | 1.00 |  |  | 0(0) | 1.01(0.99-1.02) | 1.00 |  |
| *3DL1*01502+A Bw4* | 41(24.6) |  | 33(21.3) | 0.83(0.49-1.4) | 0.49 |  |  | 44(27.2) | 1.15(0.7-1.88) | 0.59 |  |
| *3DL1*01504+A Bw4* | 0(0) |  | 0(0) | - | - |  |  | 1(0.6) | 0.99(0.98-1.01) | 0.49 |  |
| *3DL1*01505+A Bw4* | 1(0.6) |  | 0(0) | 1.01(0.99-1.02) | 1.00 |  |  | 0(0) | 1.01(0.99-1.02) | 1.00 |  |
| *3DL1*020+A Bw4* | 6(3.6) |  | 3(1.9) | 0.53(0.13-2.16) | 0.57 |  |  | 5(3.1) | 0.85(0.26-2.86) | 0.80 |  |
| *3DL1*02901+A Bw4* | 0(0) |  | 2(1.3) | 0.99(0.97-1.01) | 0.23 |  |  | 0(0) | - | - |  |
| *3DL1*077+A Bw4* | 0(0) |  | 0(0) | - | - |  |  | 1(0.6) | 0.99(0.98-1.01) | 0.49 |  |
| *3DL1*097+A Bw4* | 0(0) |  | 0(0) | - | - |  |  | 1(0.6) | 0.99(0.98-1.01) | 0.49 |  |
| *3DL2*001+A3/11* | 10(6.0) |  | 7(5.3) | 0.88(0.33-2.38) | 0.80 |  |  | 10(6.5) | 1.10(0.44-2.71) | 0.84 |  |
| *3DL2*002+A3/11* | 87(52.1) |  | 65(49.2) | 0.89(0.57-1.41) | 0.62 |  |  | 66(43.1) | 0.70(0.45-1.08) | 0.11 |  |
| *3DL2*00701+A3/11* | 1(0.6) |  | 0(0) | 1.01(0.99-1.02) | 1.00 |  |  | 1(0.7) | 1.09(0.07-17.61) | 1.00 |  |
| *3DL2*00706+A3/11* | 0(0) |  | 0(0) | - | - |  |  | 1(0.7) | 0.99(0.98-1.01) | 0.48 |  |
| *3DL2*008+A3/11* | 14(8.4) |  | 14(10.6) | 1.30(0.60-2.82) | 0.51 |  |  | 19(12.4) | 1.55(0.75-3.21) | 0.24 |  |
| *3DL2*009+A3/11* | 10(6.0) |  | 9(6.8) | 1.15(0.45-2.91) | 0.77 |  |  | 3(2) | 0.31(0.08-1.16) | 0.07 |  |
| *3DL2*010+A3/11* | 26(15.6) |  | 18(13.6) | 0.86(0.45-1.64) | 0.64 |  |  | 19(12.4) | 0.77(0.41-1.45) | 0.42 |  |
| *3DL2*015+A3/11* | 0(0) |  | 1(0.8) | 0.99(0.98-1.01) | 0.44 |  |  | 1(0.7) | 0.99(0.98-1.01) | 0.48 |  |
| *3DL2*016+A3/11* | 2(1.2) |  | 0(0) | 1.01(1.00-1.03) | 0.51 |  |  | 2(1.3) | 1.09(0.15-7.85) | 1.00 |  |
| *3DL2*021+A3/11* | 3(1.8) |  | 1(0.8) | 0.42(0.04-4.06) | 0.79 |  |  | 2(1.3) | 0.72(0.12-4.39) | 1.00 |  |
| *3DL2*036+A3/11* | 0(0) |  | 0(0) | - | - |  |  | 1(0.7) | 0.99(0.98-1.01) | 0.48 |  |
| *3DL2*039+A3/11* | 6(3.6) |  | 3(2.3) | 0.62(0.15-2.54) | 0.75 |  |  | 10(6.5) | 1.88(0.67-5.29) | 0.23 |  |
| *3DL2*063+A3/11* | 0(0) |  | 0(0) | - | - |  |  | 1(0.7) | 0.99(0.98-1.01) | 0.48 |  |
| *3DL2*083+A3/11* | 0(0) |  | 0(0) | - | - |  |  | 1(0.7) | 0.99(0.98-1.01) | 0.48 |  |
| *3DL2*084+A3/11* | 0(0) |  | 0(0) | - | - |  |  | 1(0.7) | 0.99(0.98-1.01) | 0.48 |  |
| *3DL2*091+A3/11* | 1(0.6) |  | 1(0.8) | 1.27(0.08-20.45) | 1.00 |  |  | 4(2.6) | 4.46(0.49-40.32) | 0.32 |  |

**Table S8** Comparison analysis of the observed frequencies for *KIR* allele+*HLA* interactions between healthy controls and ALL/AML patient groups having the *KIR Bx* genotype.

| KIR-HLA Ligand | Control (n=139) |  | ALL(n=163) | | | |  | AML(n=174) | | | |
| --- | --- | --- | --- | --- | --- | --- | --- | --- | --- | --- | --- |
|  | n(%) |  | n (%) | OR (95% CI) | *P* | *P_c_* |  | n (%) | OR (95% CI) | *P* | *P_c_* |
| 2DL1*001+C2 | 1(0.7) |  | 0(0) | 1.01(0.99-1.02) | 0.46 |  |  | 0(0) | 1.01(0.99-1.02) | 0.44 |  |
| 2DL1*00201+C2 | 5(3.6) |  | 7(4.3) | 1.20(0.37-3.88) | 0.76 |  |  | 10(5.7) | 1.63(0.55-4.90) | 0.38 |  |
| 2DL1*00302+C2 | 31(22.3) |  | 37(22.7) | 1.02(0.59-1.76) | 0.93 |  |  | 38(21.8) | 0.97(0.57-1.67) | 0.92 |  |
| 2DL1*00304+C2 | 1(0.7) |  | 0(0) | 1.01(0.99-1.02) | 0.46 |  |  | 0(0) | 1.01(0.99-1.02) | 0.44 |  |
| 2DL1*00305+C2 | 1(0.7) |  | 0(0) | 1.01(0.99-1.02) | 0.46 |  |  | 1(0.6) | 0.8(0.05-12.87) | 1.00 |  |
| 2DL1*00308+C2 | 0(0) |  | 0(0) | - | - |  |  | 0(0) | - | - |  |
| 2DL1*00309+C2 | 0(0) |  | 0(0) | - | - |  |  | 0(0) | - | - |  |
| 2DL1*004+C2 | 0(0) |  | 9(5.5) | 0.94(0.91-0.98) | **0.01** | 0.87 |  | 6(3.4) | 0.97(0.94-0.99) | 0.07 |  |
| 2DL1*007+C2 | 0(0) |  | 0(0) | - | - |  |  | 0(0) | - | - |  |
| 2DL1*010+C2 | 0(0) |  | 0(0) | - | - |  |  | 0(0) | - | - |  |
| 2DL1*020+C2 | 0(0) |  | 1(0.6) | 0.99(0.98-1.01) | 1.00 |  |  | 0(0) | - | - |  |
| 2DL1*030+C2 | 0(0) |  | 0(0) | - | - |  |  | 0(0) | - | - |  |
| 2DL1*031+C2 | 0(0) |  | 0(0) | - | - |  |  | 0(0) | - | - |  |
| 2DL1*033+C2 | 0(0) |  | 0(0) | - | - |  |  | 0(0) | - | - |  |
| 2DL1*034+C2 | 0(0) |  | 0(0) | - | - |  |  | 0(0) | - | - |  |
| 2DL1*069+C2 | 0(0) |  | 0(0) | - | - |  |  | 2(1.1) | 0.99(0.97-1.00) | 0.50 |  |
| 2DL1*070+C2 | 0(0) |  | 0(0) | - | - |  |  | 1(0.6) | 0.99(0.98-1.01) | 1.00 |  |
| 2DL1*071+C2 | 0(0) |  | 0(0) | - | - |  |  | 0(0) | - | - |  |
| 2DL1*072+C2 | 0(0) |  | 0(0) | - | - |  |  | 0(0) | - | - |  |
| 2DL1*073+C2 | 0(0) |  | 0(0) | - | - |  |  | 0(0) | - | - |  |
| 2DL1*074+C2 | 0(0) |  | 0(0) | - | - |  |  | 0(0) | - | - |  |
| 2DL3*00101+C1 | 120(86.3) |  | 133(81.6) | 0.70(0.38-1.31) | 0.27 |  |  | 141(81) | 0.68(0.37-1.25) | 0.21 |  |
| 2DL3*00103+C1 | 0(0) |  | 0(0) | - | - |  |  | 1(0.6) | 0.99(0.98-1.01) | 1.00 |  |
| 2DL3*00105+C1 | 0(0) |  | 0(0) | - | - |  |  | 1(0.6) | 0.99(0.98-1.01) | 1.00 |  |
| 2DL3*00108+C1 | 0(0) |  | 0(0) | - | - |  |  | 0(0) | - | - |  |
| 2DL3*00109+C1 | 3(2.2) |  | 1(0.6) | 0.28(0.03-2.72) | 0.51 |  |  | 0(0) | 1.02(1.00-1.05) | 0.17 |  |
| 2DL3*00110+C1 | 1(0.7) |  | 0(0) | 1.01(0.99-1.02) | 0.46 |  |  | 0(0) | 1.01(0.99-1.02) | 0.44 |  |
| 2DL3*00111+C1 | 0(0) |  | 0(0) | - | - |  |  | 0(0) | - | - |  |
| 2DL3*00112+C1 | 0(0) |  | 1(0.6) | 0.99(0.98-1.01) | 1.00 |  |  | 0(0) | - | - |  |
| 2DL3*00201+C1 | 26(18.7) |  | 32(19.6) | 1.06(0.60-1.89) | 0.84 |  |  | 45(25.9) | 1.52(0.88-2.61) | 0.13 |  |
| 2DL3*015+C1 | 1(0.7) |  | 3(1.8) | 2.59(0.27-25.16) | 0.73 |  |  | 4(2.3) | 3.25(0.36-29.39) | 0.51 |  |
| 2DL3*019+C1 | 0(0) |  | 1(0.6) | 0.99(0.98-1.01) | 1.00 |  |  | 0(0) | - | - |  |
| 2DL3*021+C1 | 0(0) |  | 0(0) | - | - |  |  | 0(0) | - | - |  |
| 2DL3*022+C1 | 0(0) |  | 0(0) | - | - |  |  | 2(1.1) | 0.99(0.97-1.00) | 0.50 |  |
| 2DL3*023+C1 | 3(2.2) |  | 3(1.8) | 0.85(0.17-4.28) | 1.00 |  |  | 2(1.1) | 0.53(0.09-3.2) | 0.80 |  |
| 2DL3*025+C1 | 0(0) |  | 0(0) | - | - |  |  | 0(0) | - | - |  |
| 2DL3*026+C1 | 0(0) |  | 2(1.2) | 0.99(0.97-1.00) | 0.50 |  |  | 2(1.1) | 0.99(0.97-1.00) | 0.50 |  |
| 2DL3*027+C1 | 0(0) |  | 0(0) | - | - |  |  | 2(1.1) | 0.99(0.97-1.00) | 0.50 |  |
| 2DL3*028+C1 | 0(0) |  | 0(0) | - | - |  |  | 0(0) | - | - |  |
| 2DL3*029+C1 | 0(0) |  | 0(0) | - | - |  |  | 0(0) | - | - |  |
| 2DL3*030+C1 | 0(0) |  | 1(0.6) | 0.99(0.98-1.01) | 1.00 |  |  | 0(0) | - | - |  |
| 2DL3*031+C1 | 0(0) |  | 0(0) | - | - |  |  | 0(0) | - | - |  |
| 2DL3*032+C1 | 0(0) |  | 1(0.6) | 0.99(0.98-1.01) | 1.00 |  |  | 1(0.6) | 0.99(0.98-1.01) | 1.00 |  |
| 2DL3*037+C1 | 0(0) |  | 0(0) | - | - |  |  | 0(0) | - | - |  |
| 3DL1*00101+Bw4 | 4(2.9) |  | 12(7.4) | 2.68(0.84-8.51) | 0.08 |  |  | 9(5.2) | 1.84(0.55-6.11) | 0.31 |  |
| 3DL1*00103+Bw4 | 0(0) |  | 0(0) | - | - |  |  | 0(0) | - | - |  |
| 3DL1*00401+Bw4 | 0(0) |  | 0(0) | - | - |  |  | 0(0) | - | - |  |
| 3DL1*00402+Bw4 | 0(0) |  | 0(0) | - | - |  |  | 0(0) | - | - |  |
| 3DL1*00501+Bw4 | 16(11.5) |  | 27(16.6) | 1.53(0.79-2.97) | 0.21 |  |  | 25(14.4) | 1.29(0.66-2.52) | 0.46 |  |
| 3DL1*00502+Bw4 | 0(0) |  | 0(0) | - | - |  |  | 1(0.6) | 0.99(0.98-1.01) | 1.00 |  |
| 3DL1*00701+Bw4 | 13(9.4) |  | 14(8.6) | 0.91(0.41-2.01) | 0.82 |  |  | 14(8) | 0.85(0.38-1.87) | 0.68 |  |
| 3DL1*008+Bw4 | 0(0) |  | 1(0.6) | 0.99(0.98-1.01) | 1.00 |  |  | 0(0) | - | - |  |
| 3DL1*01502+Bw4 | 57(41) |  | 75(46) | 1.23(0.78-1.94) | 0.38 |  |  | 73(42) | 1.04(0.66-1.64) | 0.87 |  |
| 3DL1*01504+Bw4 | 0(0) |  | 0(0) | - | - |  |  | 0(0) | - | - |  |
| 3DL1*01505+Bw4 | 0(0) |  | 0(0) | - | - |  |  | 0(0) | - | - |  |
| 3DL1*01508+Bw4 | 0(0) |  | 0(0) | - | - |  |  | 0(0) | - | - |  |
| 3DL1*020+Bw4 | 6(4.3) |  | 5(3.1) | 0.7(0.21-2.35) | 0.56 |  |  | 8(4.6) | 1.07(0.36-3.15) | 0.90 |  |
| 3DL1*02901+Bw4 | 4(2.9) |  | 2(1.2) | 0.42(0.08-2.32) | 0.54 |  |  | 1(0.6) | 0.2(0.02-1.77) | 0.25 |  |
| 3DL1*053+Bw4 | 0(0) |  | 0(0) | - | - |  |  | 0(0) | - | - |  |
| 3DL1*070+Bw4 | 0(0) |  | 1(0.6) | 0.99(0.98-1.01) | 1.00 |  |  | 0(0) | - | - |  |
| 3DL1*077+Bw4 | 0(0) |  | 0(0) | - | - |  |  | 2(1.1) | 0.99(0.97-1.00) | 0.50 |  |
| 3DL1*079+Bw4 | 0(0) |  | 0(0) | - | - |  |  | 0(0) | - | - |  |
| 3DL1*097+Bw4 | 0(0) |  | 1(0.6) | 0.99(0.98-1.01) | 1.00 |  |  | 2(1.1) | 0.99(0.97-1.00) | 0.50 |  |
| 3DL1*120+Bw4 | 0(0) |  | 0(0) | - | - |  |  | 0(0) | - | - |  |
| 3DL1*121+Bw4 | 0(0) |  | 0(0) | - | - |  |  | 1(0.6) | 0.99(0.98-1.01) | 1.00 |  |
| 3DL1*00101+Bw4 80I | 3(2.2) |  | 5(3.1) | 1.43(0.34-6.11) | 0.90 |  |  | 1(0.6) | 0.26(0.03-2.55) | 0.46 |  |
| 3DL1*00103+Bw4 80I | 0(0) |  | 0(0) | - | - |  |  | 0(0) | - | - |  |
| 3DL1*00401+Bw4 80I | 0(0) |  | 0(0) | - | - |  |  | 0(0) | - | - |  |
| 3DL1*00402+Bw4 80I | 0(0) |  | 0(0) | - | - |  |  | 0(0) | - | - |  |
| 3DL1*00501+Bw4 80I | 4(2.9) |  | 13(8) | 2.93(0.93-9.19) | 0.06 |  |  | 13(7.5) | 2.73(0.87-8.55) | 0.07 |  |
| 3DL1*00502+Bw4 80I | 0(0) |  | 0(0) | - | - |  |  | 0(0) | - | - |  |
| 3DL1*00701+Bw4 80I | 4(2.9) |  | 4(2.5) | 0.85(0.21-3.46) | 1.00 |  |  | 8(4.6) | 1.63(0.48-5.52) | 0.43 |  |
| 3DL1*008+Bw4 80I | 0(0) |  | 1(0.6) | 0.99(0.98-1.01) | 1.00 |  |  | 0(0) | - | - |  |
| 3DL1*01502+Bw4 80I | 19(13.7) |  | 28(17.2) | 1.31(0.7-2.47) | 0.40 |  |  | 35(20.1) | 1.59(0.86-2.93) | 0.13 |  |
| 3DL1*01504+Bw4 80I | 0(0) |  | 0(0) | - | - |  |  | 0(0) | - | - |  |
| 3DL1*01505+Bw4 80I | 0(0) |  | 0(0) | - | - |  |  | 0(0) | - | - |  |
| 3DL1*01508+Bw4 80I | 0(0) |  | 0(0) | - | - |  |  | 0(0) | - | - |  |
| 3DL1*020+Bw4 80I | 3(2.2) |  | 2(1.2) | 0.56(0.09-3.42) | 0.86 |  |  | 2(1.1) | 0.53(0.09-3.20) | 0.80 |  |
| 3DL1*02901+Bw4 80I | 2(1.4) |  | 0(0) | 1.01(0.99-1.04) | 0.21 |  |  | 0(0) | 1.01(0.99-1.04) | 0.20 |  |
| 3DL1*053+Bw4 80I | 0(0) |  | 0(0) | - | - |  |  | 0(0) | - | - |  |
| 3DL1*070+Bw4 80I | 0(0) |  | 0(0) | - | - |  |  | 0(0) | - | - |  |
| 3DL1*077+Bw4 80I | 0(0) |  | 0(0) | - | - |  |  | 0(0) | - | - |  |
| 3DL1*079+Bw4 80I | 0(0) |  | 0(0) | - | - |  |  | 0(0) | - | - |  |
| 3DL1*097+Bw4 80I | 0(0) |  | 1(0.6) | 0.99(0.98-1.01) | 1.00 |  |  | 1(0.6) | 0.99(0.98-1.01) | 1.00 |  |
| 3DL1*120+Bw4 80I | 0(0) |  | 0(0) | - | - |  |  | 0(0) | - | - |  |
| 3DL1*121+Bw4 80I | 0(0) |  | 0(0) | - | - |  |  | 1(0.6) | 0.99(0.98-1.01) | 1.00 |  |
| 3DL1*00101+Bw4 80T | 0(0) |  | 6(3.7) | 0.96(0.93-0.99) | 0.06 |  |  | 5(2.9) | 0.97(0.95-1.00) | 0.12 |  |
| 3DL1*00103+Bw4 80T | 0(0) |  | 0(0) | - | - |  |  | 0(0) | - | - |  |
| 3DL1*00401+Bw4 80T | 0(0) |  | 0(0) | - | - |  |  | 0(0) | - | - |  |
| 3DL1*00402+Bw4 80T | 0(0) |  | 0(0) | - | - |  |  | 0(0) | - | - |  |
| 3DL1*00501+Bw4 80T | 10(7.2) |  | 14(8.6) | 1.21(0.52-2.82) | 0.66 |  |  | 11(6.3) | 0.87(0.36-2.11) | 0.76 |  |
| 3DL1*00502+Bw4 80T | 0(0) |  | 0(0) | - | - |  |  | 0(0) | - | - |  |
| 3DL1*00701+Bw4 80T | 7(5) |  | 7(4.3) | 0.85(0.29-2.47) | 0.76 |  |  | 3(1.7) | 0.33(0.08-1.30) | 0.18 |  |
| 3DL1*008+Bw4 80T | 0(0) |  | 0(0) | - | - |  |  | 0(0) | - | - |  |
| 3DL1*01502+Bw4 80T | 23(16.5) |  | 35(21.5) | 1.38(0.77-2.47) | 0.28 |  |  | 34(19.5) | 1.22(0.68-2.20) | 0.50 |  |
| 3DL1*01504+Bw4 80T | 0(0) |  | 0(0) | - | - |  |  | 0(0) | - | - |  |
| 3DL1*01505+Bw4 80T | 0(0) |  | 0(0) | - | - |  |  | 0(0) | - | - |  |
| 3DL1*01508+Bw4 80T | 0(0) |  | 0(0) | - | - |  |  | 0(0) | - | - |  |
| 3DL1*020+Bw4 80T | 0(0) |  | 3(1.8) | 0.98(0.96-1.00) | 0.31 |  |  | 5(2.9) | 0.97(0.95-1.00) | 0.12 |  |
| 3DL1*02901+Bw4 80T | 1(0.7) |  | 2(1.2) | 1.71(0.15-19.11) | 1.00 |  |  | 0(0) | 1.01(0.99-1.02) | 0.44 |  |
| 3DL1*053+Bw4 80T | 0(0) |  | 0(0) | - | - |  |  | 0(0) | - | - |  |
| 3DL1*070+Bw4 80T | 0(0) |  | 0(0) | - | - |  |  | 0(0) | - | - |  |
| 3DL1*077+Bw4 80T | 0(0) |  | 0(0) | - | - |  |  | 1(0.6) | 0.99(0.98-1.01) | 1.00 |  |
| 3DL1*079+Bw4 80T | 0(0) |  | 0(0) | - | - |  |  | 0(0) | - | - |  |
| 3DL1*097+Bw4 80T | 0(0) |  | 0(0) | - | - |  |  | 1(0.6) | 0.99(0.98-1.01) | 1.00 |  |
| 3DL1*120+Bw4 80T | 0(0) |  | 0(0) | - | - |  |  | 0(0) | - | - |  |
| 3DL1*121+Bw4 80T | 0(0) |  | 0(0) | - | - |  |  | 0(0) | - | - |  |
| 3DL1*00101+A Bw4 | 2(1.4) |  | 5(3.1) | 2.17(0.41-11.35) | 0.58 |  |  | 5(2.9) | 2.03(0.39-10.61) | 0.64 |  |
| 3DL1*00103+A Bw4 | 0(0) |  | 0(0) | - | - |  |  | 0(0) | - | - |  |
| 3DL1*00401+A Bw4 | 0(0) |  | 0(0) | - | - |  |  | 0(0) | - | - |  |
| 3DL1*00402+A Bw4 | 0(0) |  | 0(0) | - | - |  |  | 0(0) | - | - |  |
| 3DL1*00501+A Bw4 | 6(4.3) |  | 7(4.3) | 0.99(0.33-3.03) | 0.99 |  |  | 11(6.3) | 1.5(0.54-4.15) | 0.44 |  |
| 3DL1*00502+A Bw4 | 0(0) |  | 0(0) | - | - |  |  | 1(0.6) | 0.99(0.98-1.01) | 1.00 |  |
| 3DL1*00701+A Bw4 | 8(5.8) |  | 8(4.9) | 0.85(0.31-2.31) | 0.74 |  |  | 9(5.2) | 0.89(0.34-2.38) | 0.82 |  |
| 3DL1*008+A Bw4 | 0(0) |  | 1(0.6) | 0.99(0.98-1.01) | 1.00 |  |  | 0(0) | - | - |  |
| 3DL1*01502+A Bw4 | 31(22.3) |  | 37(22.7) | 1.02(0.59-1.76) | 0.93 |  |  | 29(16.7) | 0.70(0.40-1.23) | 0.21 |  |
| 3DL1*01504+A Bw4 | 0(0) |  | 0(0) | - | - |  |  | 0(0) | - | - |  |
| 3DL1*01505+A Bw4 | 0(0) |  | 0(0) | - | - |  |  | 0(0) | - | - |  |
| 3DL1*01508+A Bw4 | 0(0) |  | 0(0) | - | - |  |  | 0(0) | - | - |  |
| 3DL1*020+A Bw4 | 4(2.9) |  | 3(1.8) | 0.63(0.14-2.88) | 0.83 |  |  | 5(2.9) | 1.00(0.26-3.79) | 1.00 |  |
| 3DL1*02901+A Bw4 | 3(2.2) |  | 0(0) | 1.02(1.00-1.05) | 0.19 |  |  | 1(0.6) | 0.26(0.03-2.55) | 0.46 |  |
| 3DL1*053+A Bw4 | 0(0) |  | 0(0) | - | - |  |  | 0(0) | - | - |  |
| 3DL1*070+A Bw4 | 0(0) |  | 1(0.6) | 0.99(0.98-1.01) | 1.00 |  |  | 0(0) | - | - |  |
| 3DL1*077+A Bw4 | 0(0) |  | 0(0) | - | - |  |  | 1(0.6) | 0.99(0.98-1.01) | 1.00 |  |
| 3DL1*079+A Bw4 | 0(0) |  | 0(0) | - | - |  |  | 0(0) | - | - |  |
| 3DL1*097+A Bw4 | 0(0) |  | 1(0.6) | 0.99(0.98-1.01) | 1.00 |  |  | 2(1.1) | 0.99(0.97-1.00) | 0.50 |  |
| 3DL1*120+A Bw4 | 0(0) |  | 0(0) | - | - |  |  | 0(0) | - | - |  |
| 3DL1*121+A Bw4 | 0(0) |  | 0(0) | - | - |  |  | 1(0.6) | 0.99(0.98-1.01) | 1.00 |  |
| 3DL2*001+A3/11 | 10(7.2) |  | 11(8) | 1.13(0.46-2.74) | 0.79 |  |  | 5(3) | 0.41(0.14-1.22) | 0.10 |  |
| 3DL2*002+A3/11 | 56(40.3) |  | 55(40.1) | 0.99(0.61-1.61) | 0.98 |  |  | 67(40.9) | 1.02(0.65-1.62) | 0.92 |  |
| 3DL2*003+A3/11 | 0(0) |  | 0(0) | - | - |  |  | 0(0) | - | - |  |
| 3DL2*00701+A3/11 | 36(25.9) |  | 28(20.4) | 0.73(0.42-1.29) | 0.28 |  |  | 45(27.4) | 1.08(0.65-1.80) | 0.76 |  |
| 3DL2*00702+A3/11 | 0(0) |  | 0(0) | - | - |  |  | 0(0) | - | - |  |
| 3DL2*00706+A3/11 | 1(0.7) |  | 2(1.5) | 1.71(0.15-19.11) | 1.00 |  |  | 0(0) | 1.01(0.99-1.02) | 0.44 |  |
| 3DL2*00707+A3/11 | 1(0.7) |  | 1(0.7) | 1.01(0.06-16.39) | 1.00 |  |  | 1(0.6) | 0.80(0.05-12.87) | 1.00 |  |
| 3DL2*008+A3/11 | 11(7.9) |  | 4(2.9) | 0.35(0.11-1.13) | 0.07 |  |  | 6(3.7) | 0.44(0.16-1.23) | 0.11 |  |
| 3DL2*009+A3/11 | 4(2.9) |  | 1(0.7) | 0.25(0.03-2.25) | 0.38 |  |  | 3(1.8) | 0.63(0.14-2.86) | 0.82 |  |
| 3DL2*010+A3/11 | 10(7.2) |  | 12(8.8) | 1.24(0.52-2.97) | 0.63 |  |  | 18(11) | 1.59(0.71-3.57) | 0.26 |  |
| 3DL2*01101+A3/11 | 0(0) |  | 0(0) | - | - |  |  | 0(0) | - | - |  |
| 3DL2*015+A3/11 | 3(2.2) |  | 0(0) | 1.02(1.00-1.05) | 0.19 |  |  | 0(0) | 1.02(1.00-1.05) | 0.17 |  |
| 3DL2*016+A3/11 | 0(0) |  | 0(0) | - | - |  |  | 0(0) | - | - |  |
| 3DL2*019+A3/11 | 0(0) |  | 0(0) | - | - |  |  | 0(0) | - | - |  |
| 3DL2*021+A3/11 | 2(1.4) |  | 1(0.7) | 0.50(0.05-5.62) | 1.00 |  |  | 3(1.8) | 1.28(0.21-7.75) | 1.00 |  |
| 3DL2*027+A3/11 | 2(1.4) |  | 0(0) | 1.01(0.99-1.04) | 0.21 |  |  | 0(0) | 1.01(0.99-1.04) | 0.20 |  |
| 3DL2*036+A3/11 | 0(0) |  | 0(0) | - | - |  |  | 1(0.6) | 0.99(0.98-1.01) | 1.00 |  |
| 3DL2*039+A3/11 | 5(3.6) |  | 4(2.9) | 0.81(0.21-3.07) | 1.00 |  |  | 2(1.2) | 0.33(0.06-1.73) | 0.32 |  |
| 3DL2*047+A3/11 | 0(0) |  | 0(0) | - | - |  |  | 0(0) | - | - |  |
| 3DL2*063+A3/11 | 0(0) |  | 0(0) | - | - |  |  | 0(0) | - | - |  |
| 3DL2*083+A3/11 | 0(0) |  | 0(0) | - | - |  |  | 0(0) | - | - |  |
| 3DL2*084+A3/11 | 0(0) |  | 1(0.7) | 0.99(0.98-1.01) | 1.00 |  |  | 0(0) | - | - |  |
| 3DL2*091+A3/11 | 1(0.7) |  | 1(0.7) | 1.01(0.06-16.39) | 1.00 |  |  | 1(0.6) | 0.80(0.05-12.87) | 1.00 |  |
| 3DL2*093+A3/11 | 4(2.9) |  | 3(2.2) | 0.76(0.17-3.44) | 1.00 |  |  | 8(4.9) | 1.73(0.51-5.88) | 0.37 |  |
| 3DL2*099+A3/11 | 0(0) |  | 0(0) | - | - |  |  | 0(0) | - | - |  |
